# Yiqi–Wenyang–Tiaoshen Decoction Reduces Cisplatin‐Induced Acute Kidney Injury in Rats Through Autophagy and Apoptosis Signaling Pathways Based on Network Pharmacology and Experimental Validation

**DOI:** 10.1155/mi/5435119

**Published:** 2026-05-11

**Authors:** Yunqi Bai, Yue Chang, Lili Zhang, Wenjing Zhou, Yixin Su, Jingwei Zhou

**Affiliations:** ^1^ Department of Nephrology, Dongzhimen Hospital, Beijing University of Chinese Medicine, Beijing, China, bucm.edu.cn; ^2^ Clinical Medical College, Beijing University of Chinese Medicine, Beijing, China, bucm.edu.cn; ^3^ Department of Dermatology, Beijing University of Chinese Medicine Third Affiliated Hospital, Beijing, China, bucm.edu.cn

**Keywords:** apoptosis, autophagy, cisplatin-induced acute kidney injury, network pharmacology, traditional Chinese medicine, UPLC-ESI-MS/MS

## Abstract

**Context:**

The mechanism of Yiqi–Wenyang–Tiaoshen decoction (YWT) in treating cisplatin‐induced acute kidney injury (AKI) remains unknown.

**Objective:**

This study identifies the key components of YWT and explores its therapeutic potential and mechanisms in a cisplatin‐induced AKI rat model.

**Materials and Methods:**

UPLC‐ESI‐MS/MS was utilized for the identification of compounds present in both the aqueous extract of YWT and serum samples. The overlapping components were recognized as active constituents, followed by a network pharmacological analysis. A rat model of cisplatin‐induced AKI was established, and comprehensive pathological analyses including HE, PAS, and electron microscopy, as well as biochemical assessments of serum Cre, BUN, IL‐6, and TNF‐α levels, were conducted. Western blotting was utilized to evaluate the expression levels of Caspase‐3, Caspase‐9, BAX, Bcl‐2, and LC3 Ⅱ/Ⅰ.

**Results:**

Using UPLC‐ESI‐MS/MS, we identified 182 compounds in the aqueous extract of YWT, 34 of which are confirmed to be absorbable into the bloodstream. Network pharmacological analysis suggests that YWT primarily acts by inhibiting apoptosis and activating autophagy. In the rat model, YWT significantly ameliorated renal pathology and electron microscopic features. Additionally, YWT mitigated body weight loss and renal hypertrophy while lowering serum creatinine and blood urea nitrogen levels. YWT alleviates AKI by suppressing apoptosis‐related proteins such as Caspase‐3, Caspase‐9, and BAX, enhancing Bcl‐2 expression, increasing the LC3 Ⅱ/Ⅰ ratio, and reducing p62, a marker of autophagy.

**Discussion and Conclusion:**

This study confirms the therapeutic efficacy of YWT in cisplatin‐induced AKI, potentially linked to its ability to inhibit apoptosis, activate autophagy, and mitigate mitochondrial damage.

## 1. Introduction

Cisplatin (CDDP) nephrotoxicity typically manifests as acute kidney injury (AKI) or chronic kidney damage in clinical settings, with AKI occurring in approximately 30% of cases, particularly after the initial administration and with high‐dose CDDP use [1].

Chronic kidney damage results from cumulative dosing due to multiple applications. Clinically, CDDP nephrotoxicity is characterized by proteinuria, hematuria, glycosuria, elevated serum creatinine (Scr), increased blood urea nitrogen (BUN) levels, and electrolyte imbalances, including hypomagnesemia, hypokalemia, hyponatremia, and hypocalcemia [2–4]. Current preventive and therapeutic strategies for CDDP‐induced renal damage include hemodialysis, aggressive hydration diuresis, and magnesium supplementation. Although hemodialysis effectively removes metabolites and regulates electrolyte balance, it can cause side effects such as hypophosphatemia, and arrhythmias and is associated with high costs and complex procedures [5]. Aggressive hydration diuresis is a safer and more affordable option but has suboptimal clinical efficacy [5]. Cimetidine, an H_2_ receptor blocker, provides limited protection against CDDP‐induced renal damage through OCT2 inhibition, benefiting only 14.6% of patients [6]. Carvedilol has shown protective effects in animal studies, though its clinical efficacy remains unconfirmed [7]. Sitagliptin, extensively studied, shows effectiveness against drug‐induced nephrotoxicity by reducing ROS production and inhibiting apoptosis, but large randomized controlled trials (RCTs) are still needed for confirmation [8, 9]. Given the limitations of current treatments, developing new drugs for CDDP‐induced AKI is crucial. Apoptosis, oxidative stress, and autophagy‐mediated mitochondrial damage play significant roles in the pathogenesis of AKI. CDDP activates both intrinsic and extrinsic apoptotic pathways [10, 11], promotes oxidative stress leading to excessive ROS accumulation, and causes mitochondrial dysfunction [12]. Research by Lin et al. [13] suggests that activating mitochondrial autophagy can reduce CDDP‐induced AKI. Therefore, strategies to inhibit apoptosis, reduce oxidative stress, and activate autophagy are essential for the prevention and treatment of AKI.

YWT is a traditional Chinese medicine formula developed from the clinical expertise of renowned Chinese physicians. Studies have found that this decoction can enhance chemotherapy tolerance and improve physical condition during peri‐chemotherapy period [14, 15]. Additionally, it has been shown to effectively prevent and treat CDDP‐induced AKI. Nonetheless, the exact mechanism of action of YWT in preventing and treating CDDP‐induced AKI remains unclear. Ultra Performance Liquid Chromatography‐Electrospray Ionization‐Tandem Mass Spectrometry (UPLC‐ESI‐MS/MS) is an advanced analytical technique combining the separation power of liquid chromatography with the high sensitivity and selectivity of triple quadrupole mass spectrometry (MS). It is widely used in pharmacological analysis and metabolomic profiling. Various herb extracts, specific compounds, and traditional Chinese medicine formulations have been shown to mitigate CDDP‐induced renal damage. For instance, naringin [16], icariin [17, 18], quercetin [19, 20], baicalein [21, 22], genosides [23], total saponins of *Panax notoginseng* [24], and astragaloside IV have demonstrated significant potential in preventing and treating CDDP‐induced AKI [25, 26].

This study revealed the key compounds, central targets, and primary pathways of YWT in AKI treatment through UPLC‐ESI‐MS/MS and network pharmacology analysis. It found that apoptosis and autophagy are the principal biological processes involved in the treatment of AKI, with the apoptosis pathway identified as the key pathway. Further, in vivo experiments validated the key targets of these biological processes (such as Caspase‐3, Caspase‐9, BAX, Bcl‐2, LC3 Ⅱ/Ⅰ, and p62), demonstrating that YWT exerts a significant regulatory effect on apoptosis and autophagy in CDDP‐induced AKI rats.

## 2. Materials and Methods

### 2.1. Experimental Reagents

CDDP (Beijing Aichima Biotechnology Co., Ltd., China, Lot Number: C6647‐100 mg). Hematoxylin Staining Solution, Eosin Solution (Beijing Zhongshan Golden Bridge Biotechnology Co., Ltd., China, ZLI‐9610, ZLI‐9613). Bax (50599‐2‐Ig), Bcl‐2 (26593‐1‐AP), LC3 (14600‐1‐AP), Caspase‐3 (19677‐1‐AP), Caspase‐9 (10380‐1‐AP), p62 (18420‐1‐AP), and GAPDH (10494‐1‐AP), all from Proteintech Group, Inc., USA; BCA Protein Concentration Assay Kit (Shanghai Beyotime Biotechnology Co., Ltd., China, Lot Number: B0802); PVDF Membrane (Millipore Company, USA, Lot Number: K4SA1716L); SDS‐PAGE Gel Preparation Kit (Shanghai Epizyme Biomedical Technology Co., Ltd., Lot Number: PG112); ECL Chemiluminescence Kit (Beijing Pulilai Gene Technology Co., Ltd., China, Lot Number: P1010); RIPA Lysis Buffer (Beijing Pullelai Gene Technology Co., Ltd., China, Lot Number: CI1053), and Milk Powder (Beijing Pullelai Gene Technology Co., Ltd., China, Lot Number: P1622). Microplate Reader (BIO‐TEX Instruments, Inc., USA, Model: ELX800); Vertical Electrophoresis Apparatus (Beijing Liuyi Biotechnology Co., Ltd., China, Model: DYCZ‐25D); Vertical Electrotransfer Apparatus (Beijing Liuyi Biotechnology Co., Ltd., China, Model: DYCZ‐40D); Gel Imaging System (Shanghai Tanon Technology Co., Ltd., China, Model: Tanon‐5200); Low‐Temperature Multipurpose Centrifuge (Eppendorf AG, Germany, Centrifuge 5424R); Fully Automatic Biochemical Analyzer (Beckman Coulter Company, USA, AU5800); Tissue Slicer (LeicaBiosystems, Germany, RM2135); and Tissue Embedding Machine (LeicaBiosystems, Germany, EG1150).

### 2.2. Experimental Animals

Thirty male Sprague‐Dawley rats, 6 weeks old (180–200 g), were purchased from Beijing Vital River Laboratory Animal Technology Co., Ltd. (SCXK (Beijing) 2021‐0006) and were raised in a specific pathogen‐free condition with the appropriate temperature at 22°C–25°C, humidity, and a 12 h light/dark cycle. The rats were given free access to water and a standard laboratory diet. The experiment was approved by the Animal Ethics Committee of Beijing University of Chinese Medicine (BUCM‐2023090509‐3238) and conducted in accordance with national and international guidelines for the care and use of laboratory animals. After a week of acclimatization, the rats were orally administered YWT at low, medium, or high doses (1.3 g/kg/d, 2.6 g/kg/d, or 5.6 g/kg/d) or 2 mL of distilled water for 4 days, followed by an intraperitoneal injection of CDDP (7 mg/kg) or 0.9% NaCl [27]. The general condition of the rats was observed after CDDP injection. 72 h after the CDDP injection, all rats were anesthetized with 3% pentobarbital. Blood samples were collected from the abdominal aorta for Scr and BUN analysis. Both kidneys were weighed and photographed after the connective tissue was removed. The right kidney was preserved in 4% paraformaldehyde for pathological examination, while the left kidney was stored in liquid nitrogen for Western blotting measurements. The kidney index was calculated as the ratio of the wet weight of the left kidney (g) to the body weight (g).

### 2.3. Preparation of YWT Water Extract and Serum Samples

YWT contains nine traditional Chinese herbs: *Astragalus membranaceus* (*Fisch*.) *Bunge*, *Rehmannia glutinosa* (*Gaert*.) *Libosch. ex Fisch. et Mey*., *Rhodiola rosea L*., *Polygonatum sibiricum Delar. ex Redoute*, *Cistanche deserticola Ma*, *Angelica sinensis* (*Oliv*.) *Diels*, *Ligusticum sinense* “*Chuanxiong*,” *Scutellaria baicalensis Georgi*, and *Smilax glabra Roxb*. Details of the composition are provided in Table 1. To prepare the YWT solution, soak the 1300 g of herbs for 30 min, then add five times the volume of water (6500 mL) and decoct for 30 min. Filter the solution, then add three times the volume of water (3900 mL) to the residue, and decoct for another 20 min. Filter again and combine the two filtrates, as well as the centrifuge. Collect the supernatant, concentrate it using a rotary evaporator, freeze‐dry, and weigh the extract, yielding 13.2%. The daily dosage of YWT in rats is calculated as follows: clinical adult daily prescription amount (130 g raw Chinese medicine/60 kg body weight) × 6.17 × extract yield (13.2%), resulting in an equivalent dose of 1.7 g/kg. The final extract was prepared for the low, medium, and high‐concentration dosage groups at concentrations of 1.7 g/kg, 3.4 g/kg, and 6.8 g/kg, respectively. This powder was dissolved in water to form three oral YWT solutions for rats, at concentrations of 1.7, 3.4, and 6.8 g/kg. The 0.34 g/mL YWT solution will be used for UPLC‐ESI‐MS/MS detection.

**Table 1:** The composition of YWT.

Chinese name	Accepted scientific name	Family	Batch number	Dosage (g)
Shengdihuang	*Rehmannia glutinosa* (Gaert.) Libosch. ex Fisch. et Mey.	Orobanaceae	2112241	15
Chuanxiong	*Ligusticum sinense* “Chuanxiong”	Umbelliferae	23072407	15
Huangqi	Astragalus membranaceus (Fisch.) Bunge	Leguminosae	23040603	30
Hongjingtian	*Rhodiola rosea* L.	Crassulaceae	23090603	20
Tufuling	*Smilax glabra* Roxb.	Smilacaceae	23072010	10
Huangqin	Scutellaria baicalensis Georgi	Skullcap	23061706	10
Roucongrong	*Cistanche deserticola* Ma	Orobanaceae	230708001	10
Huangjing	*Polygonatum sibiricum* Delar. ex Redoute	Asparagaceae	23052306	10
Danggui	*Angelica sinensis* (Oliv.) Diels	Umbelliferae	23052803	10

Six‐week‐old male SD rats were given a daily oral gavage of a 0.34 g/mL YWT solution for 7 consecutive days. Food was withheld for 12 h before sample collection (water was not restricted), and samples were taken 1 h after the final gavage. After anesthesia, blood was drawn from the retro‐orbital venous plexus and allowed to clot at room temperature for 1 h. The clotted blood was centrifuged at 3000 rpm for 15 min, and the supernatant was transferred to sterile centrifuge tubes and stored at −80°C. YWT metabolites in the serum were analyzed using UPLC‐ESI‐MS/MS [28].

### 2.4. MS and Chromatography Conditions

Measure 100 µL of YWT solution (0.34 g/mL) and add 300 µL of methanol. Vortex for 10 min, then centrifuge at 13,000 rpm for 10 min. Collect the supernatant for analysis. MS conditions: Ion source: electrospray; ESI scanning mode: positive and negative ion switching; detection mode: full MS/dd‐MS2; resolution: 70,000 (full mass), 17,500 (dd‐MS2); scan range: 100.0–1500.0 m/z; electrospray voltage: 3.2 kV (positive, negative); capillary temperature: 300°C; collision gas: high‐purity argon (≥99.999% purity); normalized collision energy (NCE): 30, 40, 60; sheath gas: nitrogen (≥99.999% purity), 40 Arb; auxiliary gas: nitrogen (≥99.999% purity), 15 Arb, 350°C; data acquisition time: 30 min. Chromatography conditions: column: AQ‐C18, 150 mm × 2.1 mm, 1.8 µm, Welch; flow rate: 0.30 mL/min; water phase: 0.1% formic acid in water (A); organic phase: methanol (B), gradient elution (0–1 min: 2% B; 1–5 min: 2%–20% B; 5–10 min: 20%–50% B; 10–15 min: 50%–80% B; 15–20 min: 80%–95% B; 20–27 min: 95% B; 27–28 min: 95%–2% B; 28–30 min: 2% B); column oven temperature: 35°C; autosampler temperature: 10°C; injection volume: 5 µL. Instrument: UltiMate 3000 RS (chromatography), Q Exactive (mass spectrometer). Data were acquired over 30 min, processed using CD 3.3 (Compound Discoverer 3.3, Thermo Fisher), and analyzed with mzCloud database search and MS structural analysis.

### 2.5. Identification of YWT Key Ingredients and Targets

First, key components of YWT were identified by overlapping chemical components detected in the YWT aqueous extract with metabolites found in rat serum. Swiss Target Prediction (https://www.sib.swiss/) was then used to predict active molecular targets of YWT by comparing the chemical structures of these components to known ligands using cross‐validation and statistical analysis.

### 2.6. Determination of Targets of CDDP‐Induced AKI Targets

Potential targets for treating CDDP‐induced AKI were identified using “CDDP‐induced AKI” as a keyword in the GeneCards database (www.genecards.org). The overlap between YWT key component targets and CDDP‐induced AKI targets was then determined as the potential targets of YWT for treating CDDP‐induced AKI.

### 2.7. Construction of the Protein–Protein Interaction Network

A protein–protein interaction (PPI) network was constructed using the STRING database v11.0 (www.STRING-db.org/). The resulting PPI network data were then imported into Cytoscape V3.8.0 for further analysis and visualization.

### 2.8. MCODE Module and ClueGO Analysis

Molecular Complex Detection (MCODE) analysis was used to identify clusters of CDDP‐induced AKI target genes with similar biological functions in the PPI network. The Cytoscape MCODE plug‐in automatically clusters nodes representing target genes in the PPI network and calculates the modularity score for each cluster. In this study, the MCODE analysis parameters were set as “Degree Cutoff = 2, Node Score Cutoff = 0.2, K‐Core = 2, Max. Depth = 100.” To understand the biological functions of hub target genes in the PPI network, Cytoscape ClueGO, and CluePedia plug‐ins were used for GO term enrichment analysis of each MCODE module. The ClueGO enrichment analysis parameters were set to “Show only Pathway with pV ≤ 0.05, Min Level = 4 and Max Level = 5 in GO Tree Interval, Min #Genes = 5 and %Genes = 7 in GO Term/Pathway Selection (#%Genes).”

### 2.9. GO and KEGG Enrichment Analysis

To explore the molecular mechanisms of YWT in treating CDDP‐induced AKI, gene ontology (GO) biological process enrichment analysis and Kyoto Encyclopedia of Genes and Genomes (KEGG) pathway functional annotation were performed using the DAVID database (https://david.ncifcrf.gov/) on the identified action targets of YWT components. A significance threshold of *p*  < 0.05 was set for the GO and KEGG analysis in this study.

### 2.10. Construction of Component–Target–Pathway Network

KEGG analysis identified the key components of YWT, common targets between YWT and CDDP‐induced AKI, and the top 20 signaling pathways. These elements were selected as nodes and visualized in a “component–target–pathway” network using Cytoscape 3.9.1.

### 2.11. HE and PAS Staining

The left kidney of each rat was fixed in 4% paraformaldehyde for 24 h, rinsed with tap water for 30 min, dehydrated through a graded ethanol series, embedded in paraffin, and sectioned into 3 μm thick continuous slices. The sections were stained following the kit instructions for HE and PAS staining. The pathological morphology of the rat kidneys was observed under an optical microscope, and images were captured from 6 randomly selected visual fields at 200× magnification. We conducted semi‐quantitative analysis of renal tissue sections by HE staining. Five sections per group were examined, with 10 randomly selected fields (× 200 magnification) evaluated per section. Histopathological assessment included the following features: loss of tubular brush border, tubular dilation, tubular necrosis, intratubular hemorrhage, tubular cast formation, neutrophil infiltration, and cortical necrosis. The specific criteria are as follows: 0 points, normal; 1 point, ≤10%, mild injury; 2 points, 11%–25%, moderate injury; 3 points, 26%–45%, severe injury; 4 points, 46%–75%, highly severe injury; 5 points, ≥76%, extensive injury [29, 30]. The scoring work was independently completed by two professional pathologists under double‐blind conditions.

### 2.12. Transmission Electron Microscope (TEM)

A 1mm^3^ section of fresh renal cortex was excised and fixed in 4% glutaraldehyde at 4°C for 24 h. The sample was rinsed three times with 0.1M phosphate buffer (PB, pH 7.4), each rinse lasting 15 min. The samples were then post‐fixed in 1% osmium tetroxide prepared with PB at room temperature for 2 h, followed by three 15‐minute rinses with PB. After dehydration in a graded ethanol series, the samples were permeated and embedded in acetone. Desired areas were located under a light microscope using toluidine blue staining, and ultrathin sections (60–80 nm) were prepared using an ultramicrotome. The sections were stained with 2% uranyl acetate in saturated ethanol for 8 min in the dark, followed by three washes with 70% ethanol and three washes with ultrapure water. The sections were then stained with a 2.6% lead citrate solution for 8 min, avoiding CO_2_ exposure, rinsed three times with ultrapure water, and gently blotted with filter paper. The sections were placed in a copper grid box and dried overnight at room temperature. Finally, images were captured using an HT7800/HT7700 TEM (Japan HITACHI). Detailed observations included podocyte foot processes, mitochondria, autophagy, lipid droplets, rough endoplasmic reticulum, and mitochondria‐associated membrane (MAM) structures. Image J software was used to measure the volume of mitochondria, and the specific methods were performed as described previously [31].

### 2.13. Serum BUN and Scr Test

Centrifuge the collected blood at 3000 r/min for 15 min, collect the supernatant serum, and measure BUN and Scr levels using an automatic biochemical analyzer.

### 2.14. ELISA Analysis

Centrifuge the collected blood at 3000 *r*/min for 15 min, collect the supernatant serum, and measure TNF‐α (MM‐0180R2) and IL‐6 (MM‐0190R2) levels using an ELISA kit (Jiangsu Meimian Industrial Co., Ltd., China).

### 2.15. Western Blot Analysis

Weigh 30 mg of renal tissue, chop and grind it on ice, and lyse it with RIPA lysis buffer for 30 min. Centrifuge the lysate at 15,000 × *g* for 20 min at 4°C, then collect the supernatant and quantify the protein concentration using a BCA kit. Prepare the protein samples with loading buffer and denature them at 100°C for 10 min. Load equal amounts of total protein onto an SDS‐PAGE gel for separation, then transfer the proteins to a nitrocellulose membrane. Block the membrane overnight at 4°C with 10% skim milk, and then incubate it with the following primary antibodies at room temperature: Caspase‐3 (1:1500 dilution), Caspase‐9 (1:1000 dilution), BAX (1:1000 dilution), Bcl‐2 (1:1000 dilution), LC3 (1:1000 dilution), and p62 (1:5000 dilution). After incubation, treat the membrane with detection reagent for 1 min at room temperature, and visualize the immunoreactive bands using chemiluminescence. Image acquisition was performed with a Tanon gel imaging system (Beijing Yuanping Hao Biotechnology Co., Ltd., China). Semi‐quantitative analysis of the Western blot results was conducted using Image J.

### 2.16. Statistical Analysis

Data were expressed as mean ± standard deviation and analyzed using SPSS 20.0. One‐way ANOVA was used to assess differences between groups, and results were visualized using GraphPad Prism 9. A *p*‐value of < 0.05 was considered statistically significant.

## 3. Results

### 3.1. Identification of YWT Components Through UPLC‐ESI‐MS/MS Analysis

The chemical components of the aqueous extract of YWT and its constituents in the blood were identified using UPLC‐ESI‐MS/MS technology. A total of 182 chemical components were identified in the YWT aqueous extract, as detailed in Table S1 and illustrated in the negative ion current diagram (Figure 1A). In the serum, 111 chemical components were identified, as shown in Table S2 and depicted in the negative ion current diagram (Figure 1B). Comparative analysis of the YWT aqueous extract and serum metabolite data revealed 34 compounds present in both, indicating their absorption into the bloodstream. These compounds include 7 fatty acyls, 5 flavonoids, 5 amino acids and their derivatives, 7 organic acids, 1 lipid, 2 amides, 1 flavanol, 1 phenolic compound, 1 benzene and its derivatives, 1 benzofuran, and 3 others, as detailed in Table 2. Table 2 provides comprehensive identification information for the compounds common to both YWT serum and aqueous extract, including retention time (RT, min), precursor ion molecular weight (Q1, Da), molecular weight (Da), molecular formula (Formula), ionization mode (M + H for positive, M − H for negative), compound name (Compounds), primary category (Class), and CAS number (CAS).

Figure 1Total ion chromatogram of YWT. (A) Negative ion mode chromatogram of YWT aqueous extract. (B) Negative ion mode chromatogram of YWT serum components.

**Figure:**
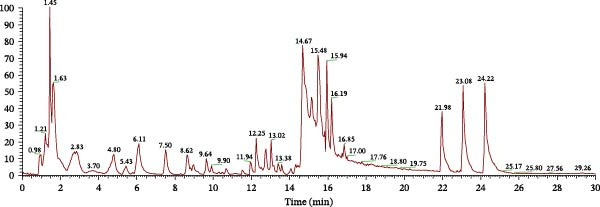
(A)

**Figure:**
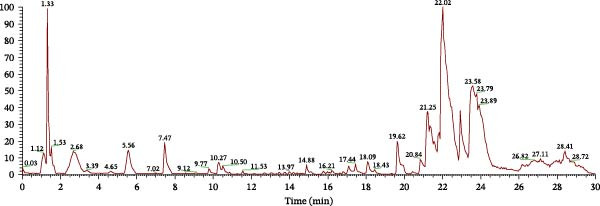
(B)

**Table 2:** Intersection of YWT aqueous extract and serum compounds.

No.	RT (min)	Q1 (Da)	Molecular weight (Da)	Formula	Ionization model	Compounds	Class	CAS
1	22.01	280.2	279.2337	C_18_H_32_O_2_	(M − H)^-^	Linoleic acid	Fatty acyls	60‐33‐3
2	7.47	204.1	188.0711	C_11_H_12_N_2_O_2_	(M + H‐NH_3_)^+^	DL‐tryptophan	Indoles and derivatives	54‐12‐6
3	14.46	446.1	447.0935	C_21_H_18_O_11_	(M + H)^+^	Baicalin	Flavonoids	21967‐41‐9
4	22.54	256.2	255.2334	C_16_H_32_O_2_	(M − H)^-^	Palmitic acid	lipids	1957‐10‐3
5	14.01	432.11	433.1130	C_21_H_20_O_10_	(M + H)^+^	Apigetrin	Flavonoids	578‐74‐5
6	22.13	304.2	305.2477	C_20_H_32_O_2_	(M + H)^+^	Arachidonic acid	Lignans and coumarins	506‐32‐1
7	5.41	170.02	169.0134	C_7_H_6_O_5_	(M − H)^−1^	Gallic acid	Phenolic acids	149‐91‐7
8	12.03	134.04	193.0500	C_10_H_10_O_4_	(M − H + HAc)^-^	Ferulic acid	Phenolic acids	1135‐24‐6
9	2.40	122.0	123.0560	C_6_H_6_N_2_O	(M + H)^+^	Nicotinamide	Others	98‐92‐0
10	17.82	234.2	235.1696	C_15_H_22_O_2_	(M + H)^+^	3,5‐di‐tert‐butyl‐4‐hydroxybenzaldehyde	Organooxygen compounds	1620‐98‐0
11	16.99	254.06	255.0651	C_15_H_10_O_4_	(M + H)^+^	Chrysin	Flavonoids	480‐40‐0
12	2.90	129.0	128.0345	C_5_H_7_NO_3_	(M − H)^-^	4‐oxoproline	Carboxylic acids and derivatives	Not available
13	14.50	476.10	477.1026	C_22_H_20_O_12_	(M + H)^+^	6‐O‐methylscutellarin	Flavonoids	31105‐76‐7
14	1.32	119.1	120.0661	C_4_H_9_NO_3_	(M + H)^+^	DL‐homoserine	Amino acids	1927‐25‐9
15	21.62	255.26	256.2635	C_16_H_33_NO	(M + H)^+^	Hexadecanamide	Fatty acyls	629‐54‐9
16	8.41	354.10	353.0880	C_16_H_18_O_9_	(M − H)^-^	Neochlorogenic acid	Polyphenolic substance	906‐33‐2
17	1.21	174.1	175.1194	C_6_H_14_N_4_O_2_	(M + H)^+^	DL‐arginine	Carboxylic acids and derivatives	7200‐25‐1
18	11.79	174.1	173.0816	C_8_H_14_O_4_	(M − H)^-^	Suberic acid	Fatty acyls	505‐48‐6
19	19.30	294.2	277.2168	C_18_H_32_O_4_	(M + H–H_2_O)^+^	(±)13‐HpODE	Fatty acyls	23017‐93‐8
20	10.40	180.04	179.0343	C_9_H_8_O_4_	(M − H)^-^	Caffeic acid	Phenolic acids	331‐39‐5
21	1.62	228.1	229.1552	C_11_H_20_N_2_O_3_	(M + H)^+^	Prolylleucine	Carboxylic acids and derivatives	Not available
22	11.93	148.05	147.0441	C_9_H_8_O_2_	(M − H)^-^	*trans*‐Cinnamic acid	Cinnamic acids and derivatives	140‐10‐3
23	1.42	196.1	195.0510	C_6_H_12_O_7_	(M − H)^-^	Gluconic acid	Others	526‐95‐4
24	11.69	120.06	119.0490	C_8_H_8_O	(M − H)^-^	Phenylacetaldehyde	Benzene and substituted derivatives	122‐78‐1
25	16.79	284.1	285.0765	C_16_H_12_O_5_	(M + H)^+^	Wogonin	Flavonoids	6665‐74‐3
26	22.94	255.26	256.2635	C_16_H_33_NO	(M + H)^+^	*N*,*N*‐diethyldodecanamide	Amides	3352‐87‐2
27	7.47	114.0	132.0813	C_9_H_9_N	(M + NH_4_)^+^	6‐methylindole	Indoles and derivatives	3420‐02‐8
28	19.34	278.2	279.2323	C_18_H_30_O_2_	(M + H)^+^	α‐Eleostearic acid	Fatty acyls	Not available
29	12.651	286.05	285.04065	C_15_ H_10_ O_6_	(M − H)^-^	Luteolin	Flavonoids	491‐70‐3
30	17.10	148.1	149.0964	C_10_H_12_O	(M + H)^+^	Cuminaldehyde	Prenol lipids	122‐03‐2
31	3.39	164.0	182.0819	C_9_H_10_O_4_	(M + NH_4_)^+^	3‐(3,4‐dihydroxyphenyl)propanoic acid	Phenylpropanoic acids	23028‐17‐3
32	16.97	194.13	195.1380	C_12_H_18_O_2_	(M + H)^+^	Sedanolide	Benzofurans	6415‐59‐4
33	15.699	166.06	167.0703	C_9_ H_10_ O_3_	(M + H)^+^	Apocynin	Phenolic compound	498‐02‐2
34	9.10	328.12	327.1089	C_15_H_20_O_8_	(M − H)^-^	4‐acetyl‐3‐hydroxy‐5‐methylphenyl β‐D‐glucopyranoside	Others	Not available

### 3.2. Acquisition of Putative Targets Between Compounds and CDDP‐Induced AKI

**Figure 2:**
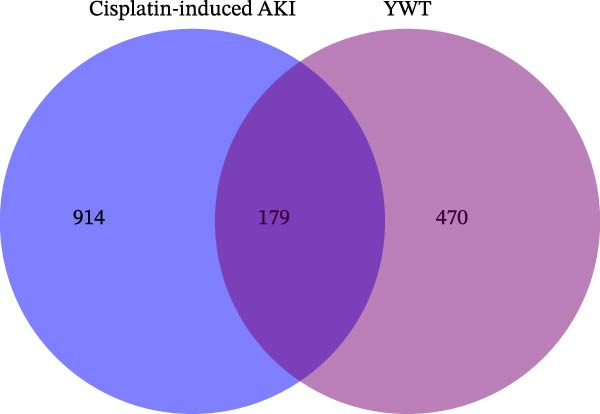
Venn diagram illustrating the overlap between cisplatin‐induced AKI targets and YWT targets.

After inputting these 34 compounds into the Swiss Target Prediction database, the predicted targets for each compound were selected as potential targets of YWT chemical components, resulting in a total of 649 unique targets after removing duplicates (Table S3). Additionally, 1093 disease targets related to CDDP‐induced AKI were retrieved from the GeneCards database (Table S4). By intersecting these with YWT targets and applying subsequent filtering, 179 common targets were identified as key targets for YWT treatment (Table S5). A Venn diagram was generated to illustrate the overlap between CDDP‐induced AKI targets and YWT targets (Figure 2).

### 3.3. Construction of PPI Network

**Figure 3:**
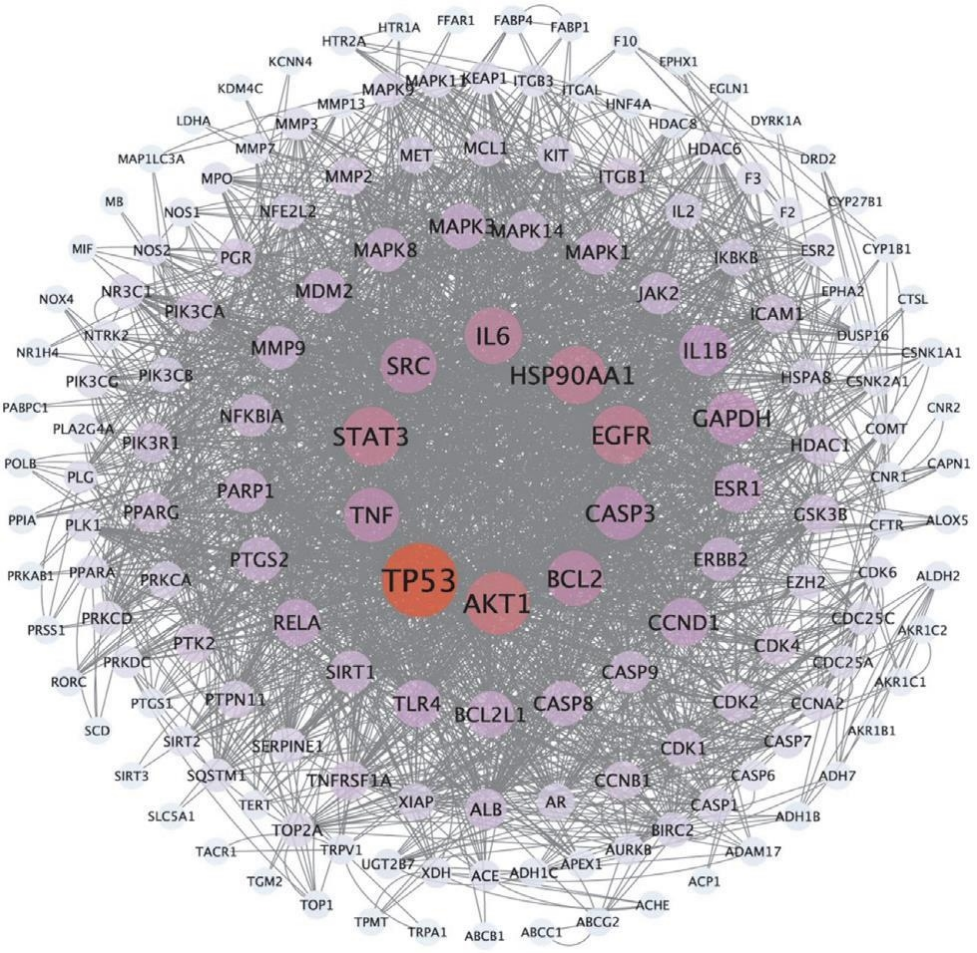
Protein–protein interaction (PPI) network of related targets for YWT in the treatment of cisplatin‐induced AKI. As the degree of targets increased, the circle became bigger, and the color changed from blue to red.

On the STRING platform (https://string-db.org/), the 179 common targets of YWT chemical components and CDDP‐induced AKI, identified through screening, were imported for interaction analysis after excluding disconnected nodes. The interaction data were exported to Cytoscape 3.9.1 for visualization, resulting in a protein–protein interaction (PPI) map for YWT in the treatment of CDDP‐induced AKI. After excluding disconnected nodes, the map contained a total of 164 nodes and 2452 edges, as shown in Figure 3.

### 3.4. GO and KEGG Analysis

GO functional annotation and KEGG pathway analysis were conducted on the 179 targets. The top 20 GO terms for biological processes, most representative from the GO enrichment analysis (adjusted *p* < 0.01), were shown in Figure 4A. The active ingredients of YWT significantly influence key processes such as response to xenobiotic stimulus, phosphorylation, regulation of apoptotic processes (both positive and negative), and regulation of autophagy.

Figure 4Go analysis (A) and KEGG enrichment (B) for YWT treatment of cisplatin‐induced AKI.

**Figure:**
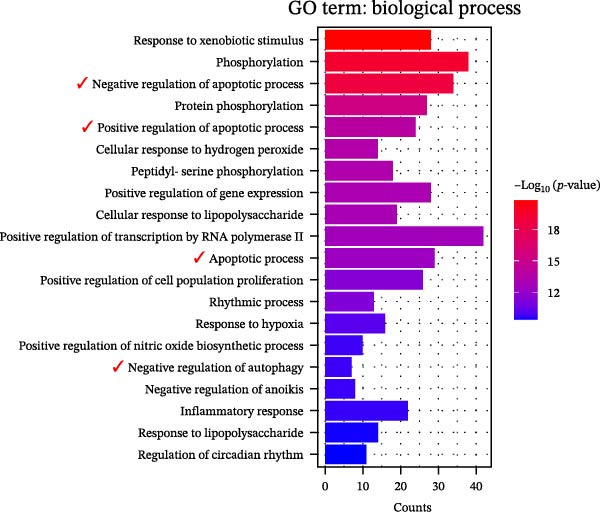
(A)

**Figure:**
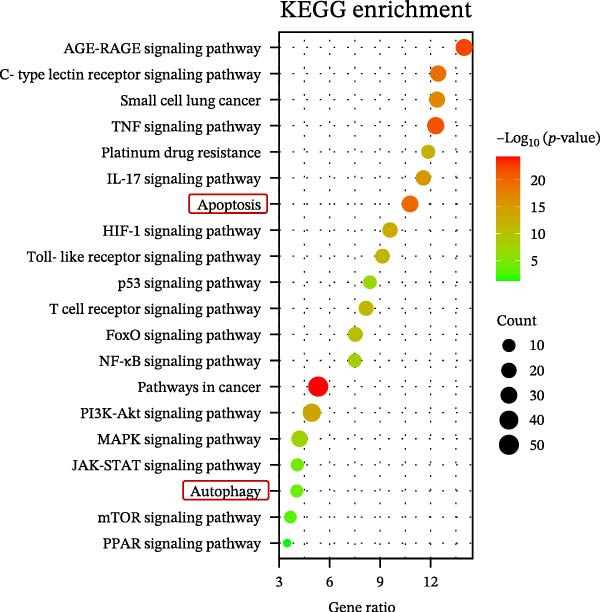
(B)

KEGG pathway analysis identified 168 significantly enriched pathways (*p* < 0.01). Figure 4B illustrates the top 20 KEGG pathways, excluding human disease pathways, including the AGE‐RAGE signaling pathway, TNF signaling pathway, apoptosis, autophagy, and mTOR signaling pathway. These results suggest that the active ingredients of YWT may mitigate CDDP‐induced AKI by modulating apoptosis and autophagy pathways.

### 3.5. Module Analysis of CDDP‐Induced AKI Targets and Their Biological Functions

Using the MCODE plugin within Cytoscape, we performed cluster module analysis on the PPI network to identify distinct biological functions of the modules. The modularity score, which reflects the degree of interconnectivity within the PPI network, identified Module 1 as the most critical functional module due to its highest modularity score. This module comprised 59 targets and 1405 edges, with a modularity score of 48.74. Key nodes in this module include BCL2, CASP3, CASP9, SQSTM1 (p62), and MAP1LC3A (LC3), as shown in Figure 5A. CluGO and CluePedia enrichment analysis indicated that the targets in Module 1 were primarily involved in biological processes such as the negative regulation of apoptosis signaling, cellular response to compound stress, and cellular response to oxidative stress, as depicted in Figure 5B.

Figure 5The MCODE module analysis identified key targets and their associated biological functions related to “cisplatin‐induced AKI.” (A) Module 1, which achieved the highest modularity score, consists of nodes where each node represents a target. (B) Each node is linked to a biological function as defined by a GO term, with the node color indicating the enrichment category of the term. Nodes sharing the same or similar biological functions are marked with the same color.

**Figure:**
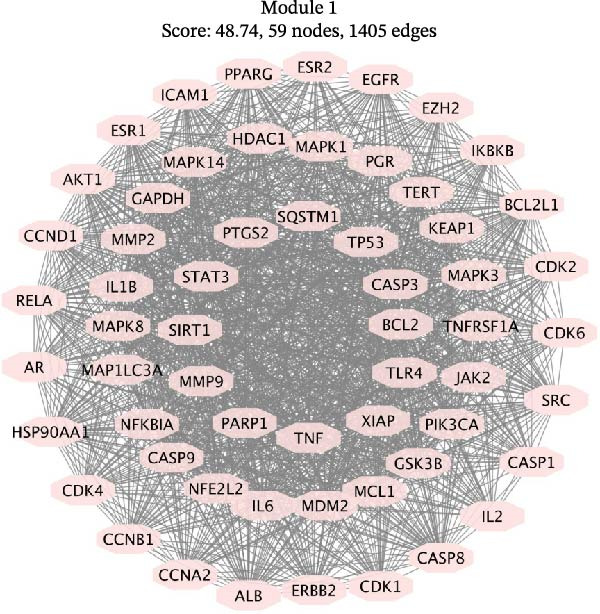
(A)

**Figure:**
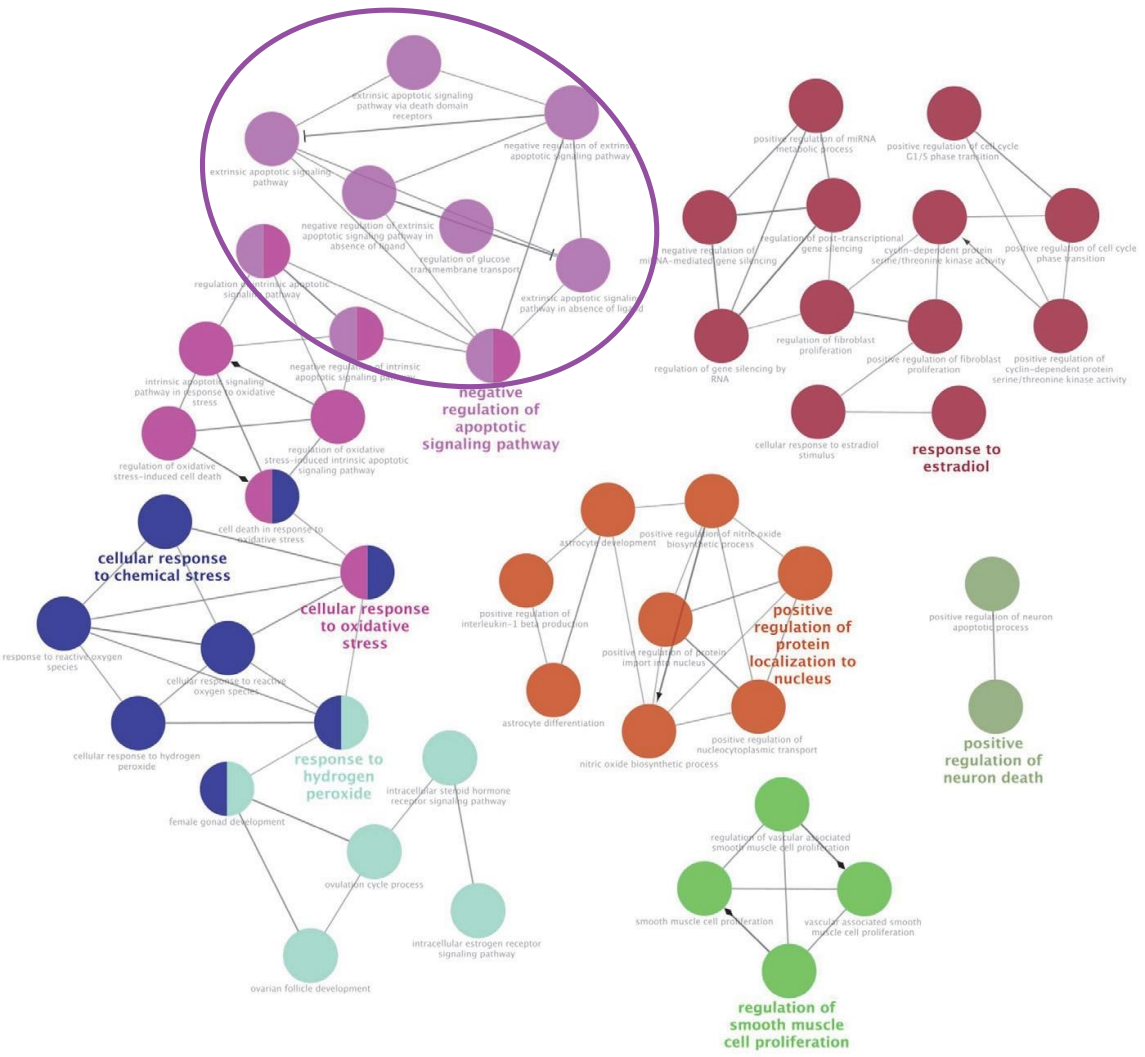
(B)

### 3.6. Compound–Target–Pathway Network Analysis

**Figure 6:**
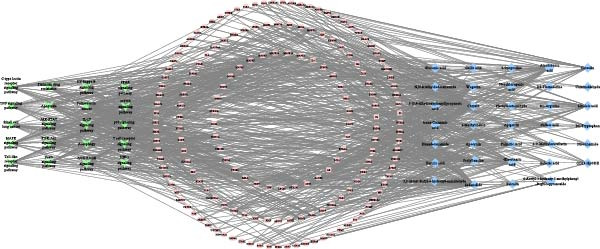
Compound–target–pathway network illustrating key compounds, targets, and the top 20 related pathways. The central circle represents the key targets, the right rhomboid nodes correspond to the key compounds in YWT, and the left polygon nodes denote the top 20 related pathways.

A compound–target–pathway network was constructed using Cytoscape 3.9.1, integrating the main compounds, key targets, and the top 20 related pathways (Figure 6). The network consists of 230 nodes and 301 edges. Among these, 15 compounds have a degree value greater than 9, including *Chrysin*, *Sedanolide*, *Wogonin*, *Arachidonic acid*,*α-Eleostearic acid*, Prolylleucine, *Linoleic acid*, *Ferulic acid*, *Toxoplasmosis*, *Luteolin*, *Palmitic acid*, *Caffeic acid*, *Suberic acid*, *DL-Tryptophan*, and (*±*)*13-HpODE*. The pathways highlighted include apoptosis, TNF signaling, mTOR signaling, and autophagy. Key targets such as BCL2, CASP3, CASP9, SQSTM1 (p62), and MAP1LC3A (LC3) are closely linked to the apoptosis signaling pathway and interact with the identified compounds. These components and targets likely represent the active ingredients and mechanisms of YWT in treating CDDP‐induced AKI.

### 3.7. YWT Alleviates CDDP‐Induced AKI

We thoroughly evaluated the protective effects of YWT on CDDP‐induced AKI by assessing rat physiological conditions, renal index, renal function indicators (Scr and BUN), and histopathological changes.

As shown in Figure 7B,C, the renal index was significantly higher in the model group compared to the control group (*p* < 0.01). However, YWT treatment at medium (YWT‐M) and high (YWT‐H) doses significantly reduced the renal index compared to the model group (*p* < 0.01). Additionally, Figure 7E,F illustrate that Scr and BUN levels were markedly elevated in the model group compared to the control group (*p* < 0.01). Treatment with YWT at low (YWT‐L), medium (YWT‐M), and high (YWT‐H) doses significantly lowered Scr and BUN levels compared to the model group (*p* < 0.05).

Figure 7(A) Schematic representation of cisplatin administration; (B) kidney index comparison among groups; (C) photographs of kidneys from different rat groups; (D) morphological photographs of rats before sampling; (E) serum BUN levels; (F) serum Scr levels; and (G) plot of weight changes in rats. Data are presented as mean ± SD (*n* = 6). ^△^ *p* < 0.01 compared to the normal group;  ^∗^ *p* < 0.05,  ^∗∗^ *p* < 0.01 compared to the model group.

**Figure:**
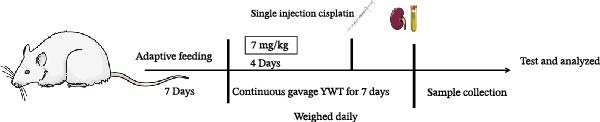
(A)

**Figure:**
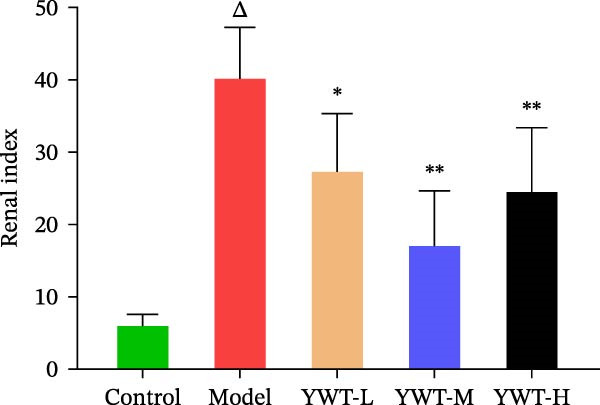
(B)

**Figure:**
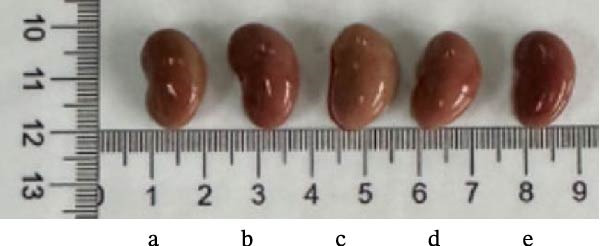
(C)

**Figure:**
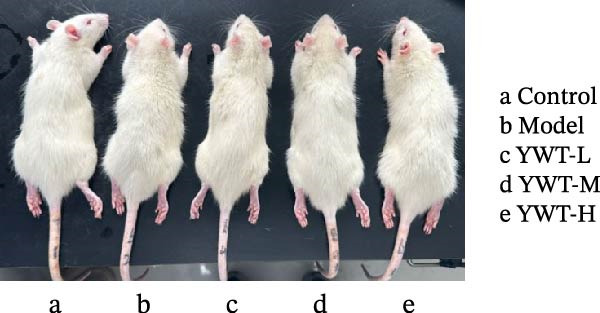
(D)

**Figure:**
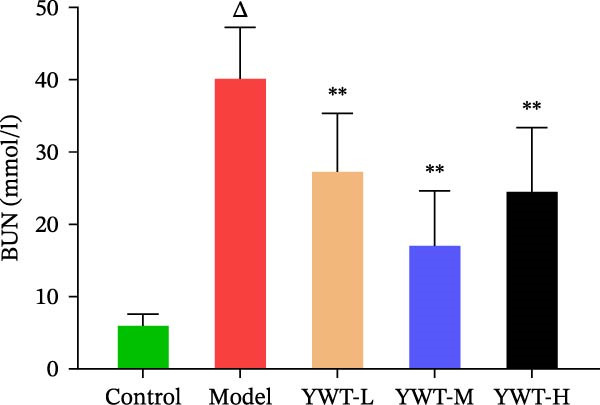
(E)

**Figure:**
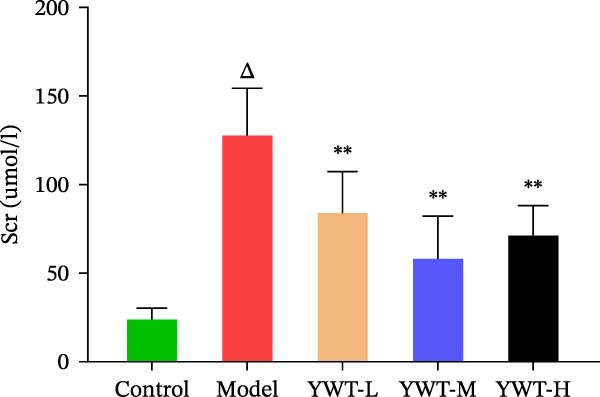
(F)

**Figure:**
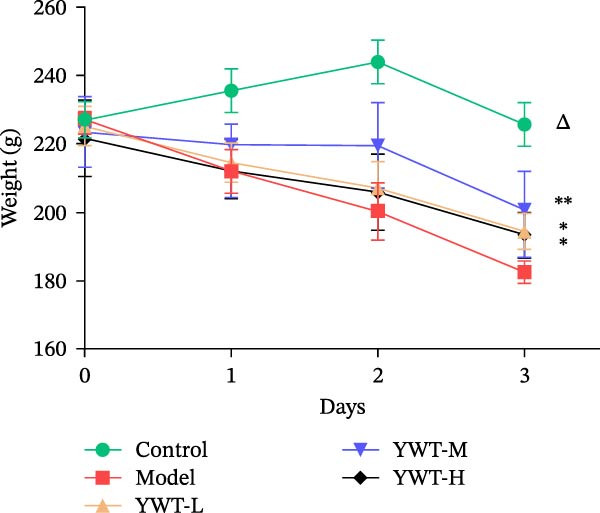
(G)

**Figure 8:**
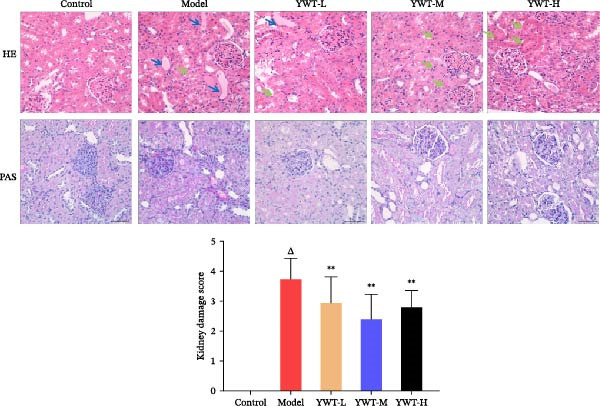
HE staining, PAS, and kidney damage score in kidneys of cisplatin‐induced AKI model rats after treatment with YWT (×200).

Histopathological analysis using HE and PAS staining (Figure 8) revealed normal renal tissue morphology in the control group, with no pathological changes. However, the model group showed marked degeneration, necrosis, and detachment of renal tubular epithelial cells, dilated renal tubules, enlarged glomerular capsule spaces, protein casts, and severe renal damage. YWT treatment at various doses significantly alleviated these pathological changes, with renal tubule morphology nearing that of the control group and reduced inflammatory cell infiltration in the renal interstitium. Overall, YWT significantly mitigated the histopathological damage in the kidneys. These results suggest that YWT can improve renal function and reduce histopathological damage in rats with CDDP‐induced AKI.

### 3.8. YWT Mitigates CDDP‐Induced AKI by Inhibiting the Inflammatory Response

Compared with the normal group, the levels of TNF‐β and IL‐6 in the model group were increased (*p*  < 0.05), and the difference was statistically significant; compared with the model group, the levels of TNF‐β and IL‐6 in the low, medium, and high doses of YWT groups decreased significantly (*p*  < 0.05), and the difference was statistically significant (Figure 9).

Figure 9Measure the levels of TNF‐α and IL‐6 using ELISA. Serum levels of TNF‐α and IL‐6 were measured by ELISA. (A) The expression level of TNF‐α in the serum. (B) The expression level of IL‐6 in the serum. Data are presented as mean ± SD (*n* = 6). ^*△*^ *p*  < 0.01, when compared with the normal group;  ^∗∗^ *p*  < 0.05, versus the model group.

**Figure:**
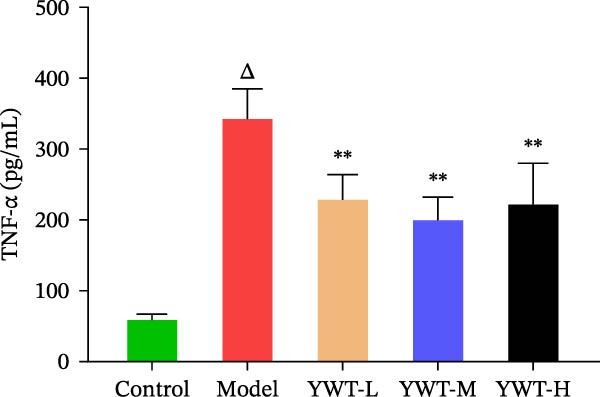
(A)

**Figure:**
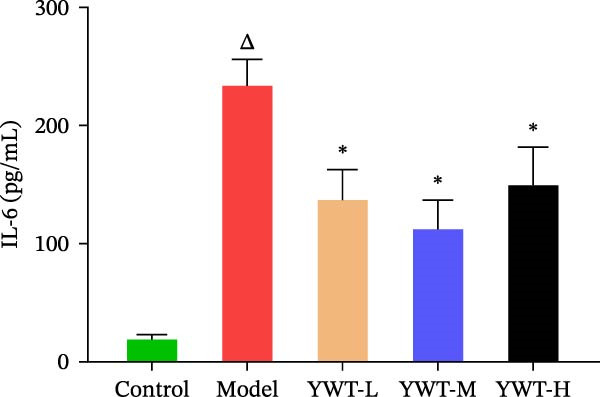
(B)

### 3.9. YWT Mitigates CDDP‐Induced AKI and Mitochondrial Damage by Activating Autophagy

CDDP inhibits autophagy, leading to adverse effects such as hepatocyte swelling, AKI, and cardiotoxicity [32]. Mitophagy, a selective form of autophagy, is crucial for degrading damaged mitochondria [33]. During the late stage of mitophagy, mitochondrial lysosomes form, a process we further evaluated using transmission electron microscopy (TEM). Our findings indicate that YWT administration reactivates autophagy suppressed by CDDP, thereby promoting the degradation of damaged mitochondria.

TEM revealed significant mitochondrial abnormalities in the CDDP treated group, including swollen mitochondria, dissolved and fragmented cristae, and reduced matrix granules. The rough endoplasmic reticulum was notably expanded into vesicular structures, with a marked increase in secondary lysosomes. Additionally, lipid droplets and autophagic structures were observed in the cytoplasm. However, treatment with YWT‐L, YWT‐M, and YWT‐H significantly ameliorated these mitochondrial damages (Figure 10).

Figure 10Transmission electron microscopy of a rat kidney model of cisplatin‐induced AKI following YWT treatment. (A,B) TEM images of renal tissues were captured. (C) Quantification of damaged mitochondria. Data are presented as mean ± SD (*n* = 6). ^△^ *p* < 0.01, when compared with normal group;  ^∗∗^ *p* < 0.01, versus model group.

**Figure:**
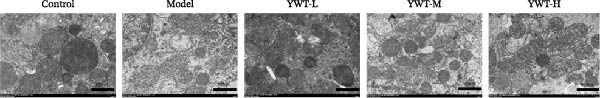
(A)

**Figure:**
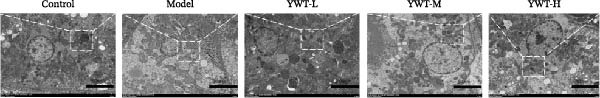
(B)

**Figure:**
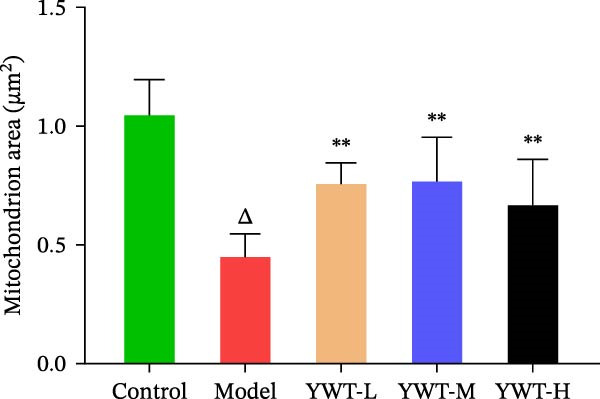
(C)

CDDP treatment resulted in a decrease in the LC3 Ⅱ/Ⅰ ratio (*p* < 0.01) (Figure 11B) and an increase in p62 expression (*p* < 0.01) (Figure 11C), indicating inhibited autophagy. Conversely, YWT‐M treatment increased the LC3 Ⅱ/Ⅰ ratio (*p* < 0.05) (Figure 11B) and decreased p62 expression (*p* < 0.05) (Figure 11C), suggesting autophagy activation.

Figure 11(A) Representative images of LC3 Ⅱ/Ⅰ and p62. The protein expression levels of (B) LC3 Ⅱ/Ⅰ ratio and (C) p62 in kidney tissue were determined using western blot (*n* = 6). Data are presented as mean ± SD (*n* = 6). ^△^ *p* < 0.01, when compared with normal group;  ^∗^ *p* < 0.05, versus model group.

**Figure:**
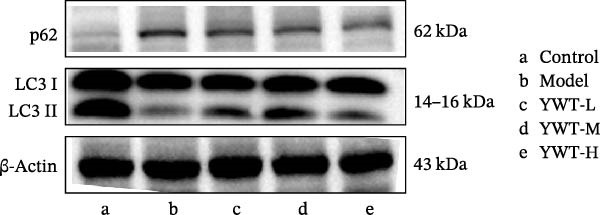
(A)

**Figure:**
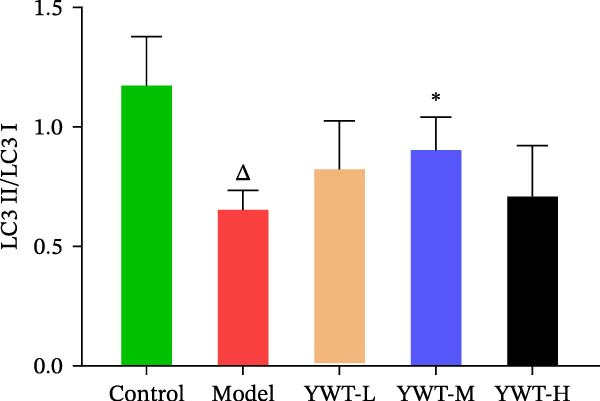
(B)

**Figure:**
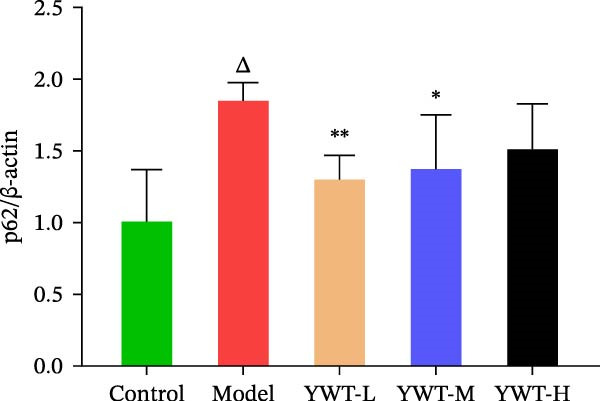
(C)

These results suggest that YWT may mitigate CDDP‐induced AKI and mitochondrial damage by activating autophagy.

### 3.10. YWT Improves CDDP‐Induced AKI by Inhibiting Apoptosis

In the model group, the levels of Caspase‐3, Caspase‐9, BAX, and the BAX/Bcl‐2 ratio were significantly elevated (*p* < 0.01), while Bcl‐2 protein expression was markedly decreased (*p* < 0.05) compared to the normal group, indicating increased apoptosis. However, treatment with low, medium, or high doses of YWT led to a significant reduction in Caspase‐3, Caspase‐9, BAX, and the BAX/Bcl‐2 ratio (*p* < 0.05 or *p*  < 0.01), alongside a significant increase in Bcl‐2 protein expression (*p* < 0.05 or *p*  < 0.01) compared to the model group (Figures 12 and 13).

Figure 12(A) Representative images of BAX, Bcl‐2, and BAX/Bcl‐2. Protein expression levels of (B) Bcl‐2, (C) BAX, (D) BAX/Bcl‐2, BAX, and Bcl‐2 in kidney tissue using Western blot (*n* = 6). Data are presented as mean ± SD (*n* = 6). ^▲^ *p* < 0.05, ^△^ *p* < 0.01, compared with normal group;  ^∗^ *p*  < 0.05,  ^∗^^∗^ *p*  < 0.01, compared with model group.

**Figure:**
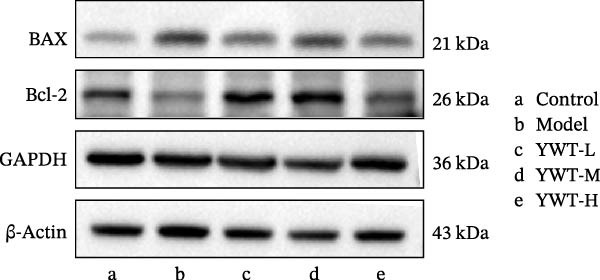
(A)

**Figure:**
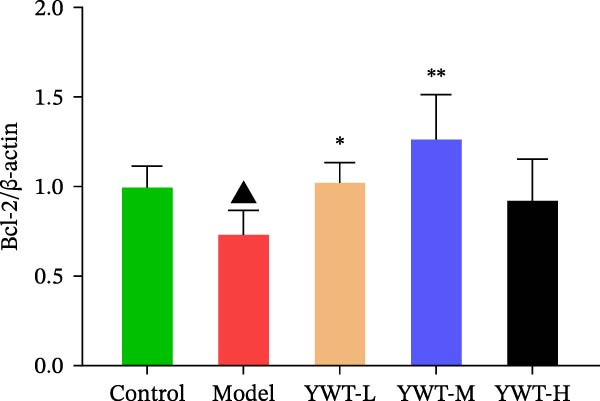
(B)

**Figure:**
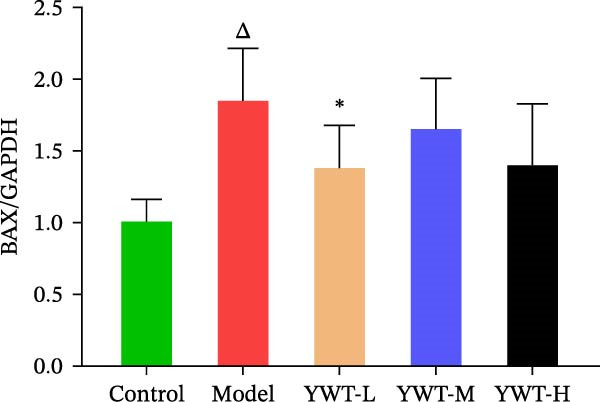
(C)

**Figure:**
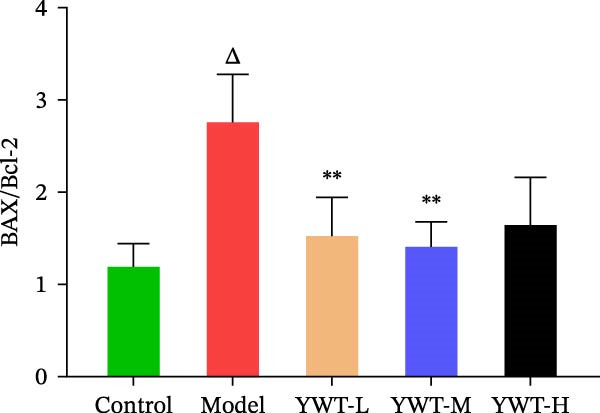
(D)

Figure 13(A) Representative images of Caspase‐3 and Caspase‐9. Protein expression levels of (B) Caspase‐3, (C) Caspase‐9, Caspase‐3, and Caspase‐9 in kidney tissue using Western blot (*n* = 6). Data are presented as mean ± SD (*n* = 6). ^△^ *p* < 0.01, compared with normal group;  ^∗^ *p* < 0.05,  ^∗^^∗^ *p*  < 0.01, compared with model group.

**Figure:**
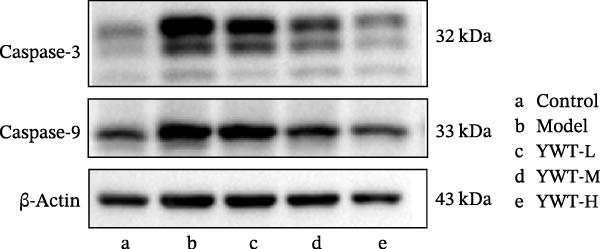
(A)

**Figure:**
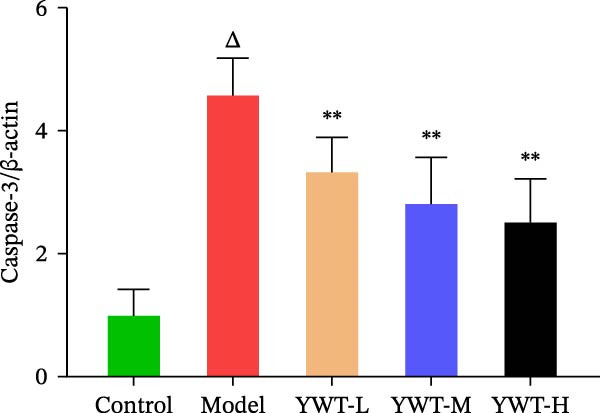
(B)

**Figure:**
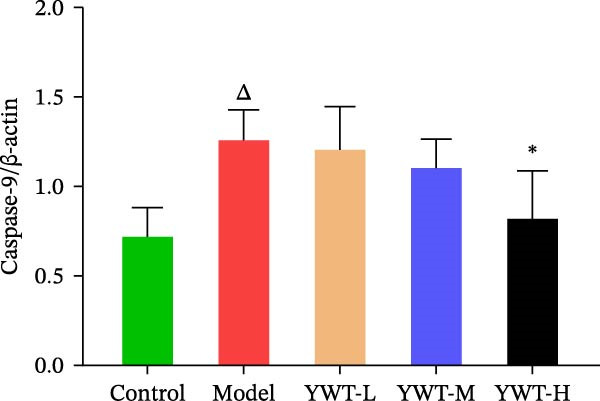
(C)

## 4. Discussion

CDDP is a widely utilized antitumor agent in clinical practice, demonstrating significant antitumor activity. It is employed in the treatment of various solid tumors and is notably effective against several malignancies, including bladder cancer and ovarian cancer [34]. CDDP is classified as a non‐cell‐cycle‐specific drug, primarily acting by covalently cross‐linking the DNA double strands of tumor cells to form platinum‐DNA adducts. This action prevents DNA replication, triggers the DNA damage response (DDR), leads to cell cycle arrest, inhibits cell proliferation, and induces cell death, thereby exerting its antitumor effects [34, 35]. However, despite its antitumor efficacy, CDDP frequently induces various adverse effects, including nephrotoxicity, gastrointestinal reactions, ototoxicity, neurotoxicity, and hematopoietic toxicity. Among these, nephrotoxicity is a major adverse effect, with approximately 30% of patients developing AKI [36], which limits its clinical application[37]. The metabolic pathway of CDDP is closely associated with its tendency to cause renal damage, as 90% of the drug is excreted in the urine, resulting in higher concentrations in the kidneys compared to other organs. In the kidneys, CDDP is primarily absorbed and excreted by transport proteins in the proximal tubules, such as OCT2 and MATE1, making the kidneys particularly susceptible to both acute and chronic kidney injury [32]. Despite extensive research over several decades, the precise mechanism underlying CDDP‐induced AKI remains incompletely understood, and effective methods for preventing and treating this adverse reaction are still lacking in clinical practice [32].

Clinical studies have demonstrated that YWT effectively mitigates AKI induced by CDDP, enhances the tolerance of cancer patients to CDDP‐based treatment regimens, and plays a role in both preventing and treating CDDP‐induced nephrotoxicity [14, 15]. This study employed a comprehensive pharmacological approach to investigate the mechanism by which YWT treats CDDP‐induced AKI. We identified 34 active components in the serum of YWT and established 179 potential targets associated with these components. Our analysis revealed that the primary effects of CDDP‐induced AKI include apoptosis and autophagy. Subsequently, we performed experimental studies to elucidate the potential mechanisms of YWT through network pharmacology analysis and validated these findings in vivo. The experimental results demonstrated that YWT significantly reduced serum levels of Scr and BUN, improved the renal index, and alleviated renal tubular damage, inflammatory cell infiltration, glomerular basement membrane thickening, and podocyte fusion, as well as mitochondrial swelling and fragmentation. These findings indicate that YWT effectively ameliorates CDDP‐induced renal dysfunction and histopathological damage.

In our UPLC‐ESI‐MS/MS analysis, we identified 182 chemical components in the aqueous extract of YWT. Similarly, in the serum samples of YWT, we identified 111 chemical components. The intersection of these two sets revealed 34 chemical components that entered the bloodstream, including 7 fatty acyls, 5 flavonoids, 5 amino acids and their derivatives, 7 organic acids, 1 lipid, 2 amides, 1 flavanol, 1 phenolic compound, 1 benzene and its derivatives, 1 benzofuran, and 3 others. Utilizing these 34 identified components as a database, network pharmacology was employed to screen for active ingredients associated with AKI induced by CDDP. Among them, 15 components exhibited a degree value greater than 9, including Chrysin, Sedanolide, Wogonin, Arachidonic acid, α‐Eleostearic acid, Prolylleucine, Linoleic acid, Ferulic acid, Toxoplasmosis, Luteolin, Palmitic acid, Caffeic acid, Suberic acid, DL‐Tryptophan, and (±)13‐HpODE. Wogonin has been shown to reduce elevated serum Scr and BUN levels following CDDP treatment by inhibiting oxidative stress, inflammation, and apoptosis, thereby alleviating tubular damage and exerting protective effects on the kidneys [38, 39]. Chrysin significantly alleviates CDDP‐induced renal damage by reducing DNA damage, inhibiting lipid peroxidation, and enhancing xanthine oxidase activity [40]. Luteolin also ameliorates CDDP‐induced renal damage by inhibiting oxidative stress and apoptosis, thereby significantly reducing tubular cell damage and improving renal function [41]. These findings suggest that YWT, containing various chemical components such as wogonin, chrysin, and luteolin, may improve CDDP‐induced AKI through the inhibition of apoptosis and autophagy.

Network pharmacology identified 179 common targets shared between YWT active ingredients and CDDP‐induced AKI. GO and KEGG functional enrichment analyses revealed that these target genes are closely associated with negative regulation of apoptosis and autophagy. In terms of apoptosis, research by Lieberthal, W. et al. found that low doses of CDDP can induce apoptosis in PTEC, while high doses can directly lead to cell necrosis [42]. CDDP‐induced apoptosis in renal tubular cells is related to the mitochondrial‐mediated intrinsic apoptotic pathway, extrinsic apoptotic pathway, and ERS pathway. CDDP can directly cause DNA damage, leading to P53 phosphorylation, which in turn leads to the activation of the pro‐apoptotic protein Bax, downregulation of the anti‐apoptotic protein Bcl‐2 expression, and promotion of the release of the apoptotic factor cytochrome C to initiate the intrinsic apoptotic pathway. Research by Jiang, M. et al. confirmed that the absence of the BAX gene can reduce apoptosis in mouse renal tubular cells [43, 44]. Both in vivo and in vitro studies have confirmed that CDDP can increase the expression of TNF and FASL in PTEC, and blocking the above pathways can reduce apoptosis in renal tubules [45, 46]. In this study, the YWT group improved mitochondrial swelling, endoplasmic reticulum structural expansion, increased secondary lysosomes, and improved renal mitochondrial damage inhibition of apoptosis. Therefore, YWT may ameliorate CDDP‐induced AKI by modulating the mitochondrial‐mediated intrinsic apoptotic pathway.

Autophagy plays a significant role in CDDP‐induced nephrotoxicity [13, 47]. Some studies have shown that activating autophagy can alleviate kidney damage [13, 48–50]. while others indicate that sustained activation of autophagy can mediate cell injury and promote renal tubulointerstitial fibrosis [51, 52]. Research by Wang, Y. et al. found that inducing mitophagy can protect against CDDP‐induced AKI both in vitro and in vivo [53]. However, Zhou, L. et al. discovered that a deficiency in PINK1 could improve CDDP‐induced AKI in rats, possibly by inhibiting DNM1L‐mediated mitochondrial fission and excessive mitophagy [54]. In this study, we found that YWT could increase the ratio of autophagy marker LC3 Ⅱ/Ⅰ (*p* < 0.05) and downregulate p62 expression (*p* < 0.05) (as shown in Figure 13), activating autophagy; electron microscopy revealed that the YWT group improved mitochondrial swelling, increased mitochondria‐lysosomes, and ameliorated mitochondrial damage in the kidneys, suggesting that YWT may improve CDDP‐induced AKI by activating mitophagy.

**Figure 14:**
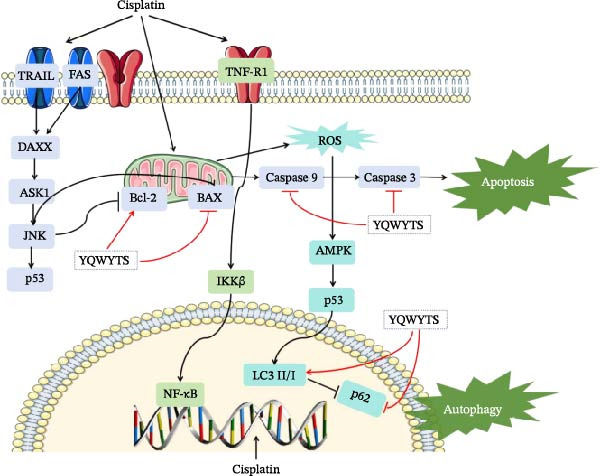
Modulating the autophagy and apoptosis signaling pathway of YWT against cisplatin‐induced AKI.

The key molecular mechanisms underlying CDDP‐induced AKI are primarily centered on apoptosis and autophagy. Network pharmacology analysis suggests that YWT significantly modulates these processes. Specifically, Caspase‐3, Caspase‐9, Bcl‐2, and BAX are critical regulators of apoptosis in CDDP‐induced AKI [42, 55]. These proteins were also identified as high‐degree targets in the component–target–disease interaction network, underscoring their importance in disease pathology. Our study demonstrated that YWT effectively decreases the expression levels of BAX, Caspase‐3, Caspase‐9, and the BAX/Bcl‐2 ratio, while increasing Bcl‐2 protein expression. Additionally, electron microscopy reveals that YWT ameliorates mitochondrial swelling, expansion of the endoplasmic reticulum, and increases in secondary lysosomes. YWT also enhances mitochondrial autophagy, as evidenced by an increased LC3 Ⅱ/Ⅰ ratio and decreased p62 levels. Thus, YWT may improve CDDP‐induced AKI by modulating the mitochondrial‐mediated intrinsic apoptosis pathway and activating mitophagy, as illustrated in Figure 14.

Despite having successfully demonstrated in animal models that YWT can alleviate CDDP‐induced AKI, with potential mechanisms involving the suppression of oxidative stress and apoptosis, as well as the activation of autophagy, the precise underlying mechanism pathways still warrant further in‐depth research. In the present study, our assessment of renal injury was solely based on Scr and BUN levels. In future research endeavors, it would be advisable to employ a broader range of methods for evaluating renal injury, encompassing indicators such as KIM‐1 and NGAL. The current study selects caspase‐3/9 activity detection as the core indicator for assessing apoptosis, mainly considering its direct correlation with the mitochondrial apoptotic pathway. To build a more comprehensive framework for assessing apoptosis, TUNEL detection will be introduced in future studies. In this study, we only used animal models for experimental verification. In the subsequent research, we will adopt multiple approaches for validation, including cell experiments. We utilized UPLC‐ESI‐MS/MS technology to identify 34 absorbable compounds from the serum. However, the question of whether there are additional compounds, along with the experimental validation of these identified compounds, remains unaddressed. Consequently, our subsequent research can place a particular emphasis on the validation of compound components and in vivo pharmacokinetic studies.

## 5. Conclusion

In conclusion, the chemotherapy drug CDDP is indispensable in antitumor therapy, and in‐depth understanding of its mechanism of renal toxicity is of great importance for the development of the emerging field of tumor nephrology. Although the current understanding of CDDP nephrotoxicity has reached the level of molecular biology, there is still a lack of effective therapeutic drugs. It is urgent to ensure the therapeutic effect for cancer patients while reducing the toxic side effects brought by treatment. Oncologists and nephrologists are still required to pay attention to this problem and escort the lives and health of patients. It is believed that with the deepening of research, certain progress will be made in the aspect of CDDP nephrotoxicity.

## Author Contributions

**Yunqi Bai, Yue Chang, and Lili Zhang:** conceptualization, data curation, validation, visualization, investigation, writing – original draft. **Wenjing Zhou**: data curation. **Yixin Su and Jingwei Zhou**: resources, project administration, supervision, reviewing and editing the manuscript draft.

## Funding

This work was supported by the Beijing University of Chinese Medicine (Grant 2023‐JYB‐900701‐014, China).

## Disclosure

All the listed authors in this manuscript give their consent to publish the manuscript.

## Ethics Statement

The experimental protocols were approved by the Beijing University of Traditional Chinese Medicine, and the certificate number is BUCM‐2023090509‐3238.

## Conflicts of Interest

The authors declare no conflicts of interest.

## Supporting Information

Additional supporting information can be found online in the Supporting Information section.

## Supporting information


**Supporting Information** Table S1: Identified solution compounds of YWT by UPLC‐MS/MS. Table S2: Identified serum compounds of YWT by UPLC‐MS/MS. Table S3: The targets of YWT. Table S4: The targets of cisplatin‐induced AKI. Table S5: The key targets of YWT in cisplatin‐induced AKI.

## Data Availability

Following the publication of the article, the data utilized in our research findings will be available upon request by contacting the authors.

## References

[1] Kociba R. J. and Sleight S. D. , Acute Toxicologic and Pathologic Effects of Cis-Diamminedichloroplatinum (NSC-119875) in the Male Rat, Cancer Chemotherapy Reports. (1971) 55, no. 1, 1–8. 5121647

[2] Greggi Antunes L. M. , Darin J. D. C. , and Bianchi M. D. L. P. , Effects of the Antioxidants Curcumin or Selenium on Cisplatin-Induced Nephrotoxicity and Lipid Peroxidation in Rats, Pharmacological Research. (2001) 43, no. 2, 145–150, 10.1006/phrs.2000.0724, 2-s2.0-0034959846. 11243715

[3] Goren M. P. , Cisplatin Nephrotoxicity Affects Magnesium and Calcium Metabolism, Medical and Pediatric Oncology. (2003) 41, no. 3, 186–189, 10.1002/mpo.10335, 2-s2.0-0042844496. 12868117

[4] Ali B. H. , Al-Moundhri M. , and Tageldin M. , et al.Ontogenic Aspects of Cisplatin-Induced Nephrotoxicity in Rats, Food and Chemical Toxicology. (2008) 46, no. 11, 3355–3359, 10.1016/j.fct.2008.07.030, 2-s2.0-54449089619. 18790000

[5] Crona D. J. , Faso A. , Nishijima T. F. , McGraw K. A. , Galsky M. D. , and Milowsky M. I. , A Systematic Review of Strategies to Prevent Cisplatin-Induced Nephrotoxicity, The Oncologist. (2017) 22, no. 5, 609–619, 10.1634/theoncologist.2016-0319, 2-s2.0-85019065939. 28438887 PMC5423518

[6] jing Z. , Effects of Gene Polymorphism, Cimetidine and Sex on Cisplatin Nephrotoxicity, 2012, Shandong University.

[7] Esmaeeli A. , Keshavarz Z. , Dehdar F. , Assadi M. , and Seyedabadi M. , The Effects of Carvedilol, Metoprolol and Propranolol on Cisplatin-Induced Kidney Injury, Drug and Chemical Toxicology. (2022) 45, no. 4, 1558–1564, 10.1080/01480545.2020.1846551. 33198524

[8] Shayan M. and Elyasi S. , Cilastatin as a Protective Agent Against Drug-Induced Nephrotoxicity: A Literature Review, Expert Opinion on Drug Safety. (2020) 19, no. 8, 999–1010, 10.1080/14740338.2020.1796967. 32666842

[9] Zaballos M. , et al.Effect of Cilastatin on Cisplatin-Induced Nephrotoxicity in Patients Undergoing Hyperthermic Intraperitoneal Chemotherapy, International Journal of Molecular Sciences. (2021) 22, no. 3, 10.3390/ijms22031239, 1239. 33513824 PMC7865672

[10] Fuertes M. , Castilla J. , Alonso C. , and Pérez J. , Cisplatin Biochemical Mechanism of Action: From Cytotoxicity to Induction of Cell Death Through Interconnections Between Apoptotic and Necrotic Pathways, Current Medicinal Chemistry. (2003) 10, no. 3, 257–266, 10.2174/0929867033368484, 2-s2.0-12244262808. 12570712

[11] Tchounwou P. B. , Dasari S. , Noubissi F. K. , Ray P. , and Kumar S. , Advances in Our Understanding of the Molecular Mechanisms of Action of Cisplatin in Cancer Therapy, Journal of Experimental Pharmacology. (2021) 13, 303–328, 10.2147/JEP.S267383. 33776489 PMC7987268

[12] Kim R. , Tanabe K. , Uchida Y. , Emi M. , Inoue H. , and Toge T. , Current Status of the Molecular Mechanisms of Anticancer Drug-Induced Apoptosis, Cancer Chemotherapy and Pharmacology. (2002) 50, no. 5, 343–352, 10.1007/s00280-002-0522-7, 2-s2.0-0036449793. 12439591

[13] Lin Q. , Li S. , and Jin H. , et al.Mitophagy Alleviates Cisplatin-Induced Renal Tubular Epithelial Cell Ferroptosis Through ROS/HO-1/GPX4 Axis, International Journal of Biological Sciences. (2023) 19, no. 4, 1192–1210, 10.7150/ijbs.80775. 36923942 PMC10008689

[14] jinming X. and shijie Z. , Treatment of 30 Cases of Chemotherapy Side Effects of Senile Malignant Tumor With Yigengyang Jianpi Decoction, Journal of Chinese Medicine. (2012) 53, 2.

[15] Peng X. , Effect Evaluation and Mechanism Study of Yiqengyang Prescription in Improving Physical State During Perichemotherapy, 2019, Beijing University of Chinese Medicine.

[16] Chtourou Y. , Aouey B. , Aroui S. , Kebieche M. , and Fetoui H. , Anti-Apoptotic and Anti-Inflammatory Effects of Naringin on Cisplatin-Induced Renal Injury in the Rat, Chemico-Biological Interactions. (2016) 243, 1–9, 10.1016/j.cbi.2015.11.019, 2-s2.0-84948699781. 26612654

[17] Ma P. , Zhang S. , Su X. , Qiu G. , and Wu Z. , Protective Effects of Icariin on Cisplatin-Induced Acute Renal Injury in Mice, American Journal of Translational Research. (2014) 7, 2105–2114. PMC4656788 26692955

[18] Zhou Y.-D. , Hou J.-G. , and Yang G. , et al.Icariin Ameliorates Cisplatin-Induced Cytotoxicity in Human Embryonic Kidney 293 Cells by Suppressing ROS-Mediated PI3K/Akt Pathway, Biomedicine & Pharmacotherapy. (2019) 109, 2309–2317, 10.1016/j.biopha.2018.11.108, 2-s2.0-85057217990. 30551489

[19] Muñoz-Reyes D. , Casanova A. G. , and Tascón J. , et al.Role of Quercetin Metabolites in the Flavonoid?s Protective Effect Against Cisplatin Nephrotoxicity, Toxicology Letters. (2021) 350, 10.1016/S0378-4274(21)00478-1, S98.

[20] Shi M. , Mobet Y. , and Shen H. , Quercetin Attenuates Acute Kidney Injury Caused by Cisplatin by Inhibiting Ferroptosis and Cuproptosis, Cell Biochemistry and Biophysics. (2024) 82, no. 3, 2687–2699, 10.1007/s12013-024-01379-6. 39026057

[21] Awadalla A. , Mahdi M. R. , and Zahran M. H. , et al.Baicalein and Alpha-Tocopherol Inhibit Toll-Like Receptor Pathways in Cisplatin-Induced Nephrotoxicity, Molecules. (2022) 27, no. 7, 10.3390/molecules27072179, 2179. 35408581 PMC9000769

[22] Liu S. , Gao X. , and Wang Y. , et al.Baicalein-Loaded Silk Fibroin Peptide Nanofibers Protect Against Cisplatin-Induced Acute Kidney Injury: Fabrication, Characterization and Mechanism, International Journal of Pharmaceutics. (2022) 626, 10.1016/j.ijpharm.2022.122161, 122161. 36058409

[23] Luo X. , Xie D. , Chen Z. , and Ji Q. , Protective Effects of Ginsenosides in Cisplatin-Induced Kidney Injury: A Systematic Review, Meta-Analysis, Indian Journal of Pharmacology. (2023) 55, no. 4, 243–250, 10.4103/ijp.ijp_251_23. 37737077 PMC10657623

[24] Li Q. , Liang X. , and Yang Y. , et al. *Panax notoginseng* Saponins Ameliorate Cisplatin-Induced Mitochondrial Injury via the HIF-1α/Mitochondria/ROS Pathway, FEBS Open Bio. (2020) 10, no. 1, 118–126, 10.1002/2211-5463.12760. PMC6943232 31715069

[25] Yan W. , Xu Y. , and Yuan Y. , et al.Renoprotective Mechanisms of Astragaloside IV in Cisplatin-Induced Acute Kidney Injury, Free Radical Research. (2017) 51, no. 7-8, 669–683, 10.1080/10715762.2017.1361532, 2-s2.0-85027350320. 28750561

[26] Qu X. , Gao H. , and Tao L. , et al.Astragaloside IV Protects against Cisplatin-Induced Liver and Kidney Injury via Autophagy-Mediated Inhibition of NLRP3 in Rats, The Journal of Toxicological Sciences. (2019) 44, no. 3, 167–175, 10.2131/jts.44.167, 2-s2.0-85062593278. 30842369

[27] Sami D. H. , Soliman A. S. , and Khowailed A. A. , et al.7-Hydroxycoumarin Modulates Nrf2/HO-1 and MicroRNA-34a/SIRT1 Signaling and Prevents Cisplatin-Induced Oxidative Stress, Inflammation, and Kidney Injury in Rats, Life Sciences. (2022) 310, 10.1016/j.lfs.2022.121104, 121104. 36270424

[28] Fraga C. G. , Clowers B. H. , Moore R. J. , and Zink E. M. , Signature-Discovery Approach for Sample Matching of a Nerve-Agent Precursor Using Liquid Chromatography−Mass Spectrometry, XCMS, and Chemometrics, Analytical Chemistry. (2010) 82, no. 10, 4165–4173, 10.1021/ac1003568, 2-s2.0-77952498181. 20405949

[29] Deng Z. , Sun M. , and Wu J. , et al.SIRT1 Attenuates Sepsis-Induced Acute Kidney Injury via Beclin1 Deacetylation-Mediated Autophagy Activation, Cell Death & Disease. (2021) 12, no. 2, 10.1038/s41419-021-03508-y, 217. 33637691 PMC7910451

[30] Jiang Y. , Zeng Y. , and Huang X. , et al.Nur77 Attenuates Endothelin-1 Expression via Downregulation of NF-κB and p38 MAPK in A549 Cells and in an ARDS Rat Model, American Journal of Physiology-Lung Cellular and Molecular Physiology. (2016) 311, no. 6, L1023–L1035, 10.1152/ajplung.00043.2016, 2-s2.0-85006062144. 27765761 PMC5206403

[31] Gong Q. , Lai T. , Liang L. , Jiang Y. , and Liu F. , Targeted Inhibition of CX3CL1 Limits Podocytes Ferroptosis to Ameliorate Cisplatin-Induced Acute Kidney Injury, Molecular Medicine. (2023) 29, no. 1, 10.1186/s10020-023-00733-3, 140. 37875838 PMC10594885

[32] Oh G.-S. , Kim H.-J. , and Shen A. H. , et al.Cisplatin-Induced Kidney Dysfunction and Perspectives on Improving Treatment Strategies, Electrolytes & Blood Pressure. (2014) 12, no. 2, 10.5049/EBP.2014.12.2.55, 2-s2.0-84921688340, 55. 25606044 PMC4297704

[33] Fu Z.-J. , Wang Z.-Y. , and Xu L. , et al.HIF-1α-BNIP3-Mediated Mitophagy in Tubular Cells Protects Against Renal Ischemia/Reperfusion Injury, Redox Biology. (2020) 36, 10.1016/j.redox.2020.101671, 101671. 32829253 PMC7452120

[34] Gandin V. , Hoeschele J. D. , and Margiotta N. , Special Issue Cisplatin in Cancer Therapy: Molecular Mechanisms of Action 3.0, International Journal of Molecular Sciences. (2023) 24, no. 9, 10.3390/ijms24097917, 7917. 37175624 PMC10178266

[35] Klumpers M. J. , Witte W. D. , and Gattuso G. , et al.Genome-Wide Analyses of Nephrotoxicity in Platinum-Treated Cancer Patients Identify Association With Genetic Variant in RBMS3 and Acute Kidney Injury, Journal of Personalized Medicine. (2022) 12, no. 6, 10.3390/jpm12060892, 892. 35743677 PMC9224783

[36] Latcha S. , Jaimes E. A. , Patil S. , Glezerman I. G. , Mehta S. , and Flombaum C. D. , Long–Term Renal Outcomes after Cisplatin Treatment, Clinical Journal of the American Society of Nephrology. (2016) 11, no. 7, 1173–1179, 10.2215/CJN.08070715, 2-s2.0-85015583179. 27073199 PMC4934839

[37] Dasari S. , Njiki S. , Mbemi A. , Yedjou C. G. , and Tchounwou P. B. , Pharmacological Effects of Cisplatin Combination With Natural Products in Cancer Chemotherapy, International Journal of Molecular Sciences. (2022) 23, no. 3, 10.3390/ijms23031532, 1532. 35163459 PMC8835907

[38] Meng X.-M. , Li H.-D. , and Wu W.-F. , et al.Wogonin Protects Against Cisplatin-Induced Acute Kidney Injury by Targeting RIPK1-Mediated Necroptosis, Laboratory Investigation. (2018) 98, no. 1, 79–94, 10.1038/labinvest.2017.115, 2-s2.0-85042776196. 29200200

[39] Badawy A. M. , El-Naga R. N. , Gad A. M. , Tadros M. G. , and Fawzy H. M. , Wogonin Pre-Treatment Attenuates Cisplatin-Induced Nephrotoxicity in Rats: Impact on PPAR-γ, Inflammation, Apoptosis and Wnt/β-Catenin Pathway, Chemico-Biological Interactions. (2019) 308, 137–146, 10.1016/j.cbi.2019.05.029, 2-s2.0-85066156419. 31103702

[40] Sultana S. , Verma K. , and Khan R. , Nephroprotective Efficacy of Chrysin against Cisplatin-Induced Toxicity via Attenuation of Oxidative Stress, Journal of Pharmacy and Pharmacology. (2012) 64, no. 6, 872–881, 10.1111/j.2042-7158.2012.01470.x, 2-s2.0-84861092471. 22571266

[41] Kang K. P. , Park S. K. , and Kim D. H. , et al.Luteolin Ameliorates Cisplatin-Induced Acute Kidney Injury in Mice by Regulation of p53-Dependent Renal Tubular Apoptosis, Nephrology Dialysis Transplantation. (2011) 26, no. 3, 814–822, 10.1093/ndt/gfq528, 2-s2.0-79952154960. 20817674

[42] Lieberthal W. , Triaca V. , and Levine J. , Mechanisms of Death Induced by Cisplatin in Proximal Tubular Epithelial Cells: Apoptosis vs. Necrosis, American Journal of Physiology-Renal Physiology. (1996) 270, no. 4, F700–F708, 10.1152/ajprenal.1996.270.4.F700. 8967349

[43] Jiang M. , Wang C.-Y. , Huang S. , Yang T. , and Dong Z. , Cisplatin-Induced Apoptosis in p53-Deficient Renal Cells via the Intrinsic Mitochondrial Pathway, American Journal of Physiology-Renal Physiology. (2009) 296, no. 5, F983–F993, 10.1152/ajprenal.90579.2008, 2-s2.0-66049128092. 19279129 PMC2681364

[44] Fan X. , Wei W. , Huang J. , Liu X. , and Ci X. , Isoorientin Attenuates Cisplatin-Induced Nephrotoxicity Through the Inhibition of Oxidative Stress and Apoptosis via Activating the SIRT1/SIRT6/Nrf-2 Pathway, Frontiers in Pharmacology. (2020) 11, 10.3389/fphar.2020.00264, 264. 32256355 PMC7093647

[45] Tsuruya K. , Ninomiya T. , and Tokumoto M. , et al.Direct Involvement of the Receptor-Mediated Apoptotic Pathways in Cisplatin-Induced Renal Tubular Cell Death, Kidney International. (2003) 63, no. 1, 72–82, 10.1046/j.1523-1755.2003.00709.x, 2-s2.0-6444245177. 12472770

[46] Linkermann A. , Himmerkus N. , and Rölver L. , et al.Renal Tubular Fas Ligand Mediates Fratricide in Cisplatin-Induced Acute Kidney Failure, Kidney International. (2011) 79, no. 2, 169–178, 10.1038/ki.2010.317, 2-s2.0-78650875224. 20811331

[47] Dil E. , Topcu A. , and Mercantepe T. , et al.Agomelatine on Cisplatin-Induced Nephrotoxicity via Oxidative Stress and Apoptosis, Naunyn-Schmiedeberg’s Archives of Pharmacology. (2023) 396, no. 10, 2753–2764, 10.1007/s00210-023-02632-0. 37480488

[48] Shi M. , Maique J. , and Shepard S. , et al.In Vivo Evidence for Therapeutic Applications of beclin 1 to Promote Recovery and Inhibit Fibrosis After Acute Kidney Injury, Kidney International. (2022) 101, no. 1, 63–78, 10.1016/j.kint.2021.09.030. 34736972 PMC8741729

[49] Shi L. , Song Z. , and Li C. , et al.HDAC6 Inhibition Alleviates Ischemia- and Cisplatin-Induced Acute Kidney Injury by Promoting Autophagy, Cells. (2022) 11, no. 24, 10.3390/cells11243951, 3951. 36552715 PMC9776591

[50] Yuan Y. , Yuan L. , and Yang J. , et al.Autophagy-Deficient Macrophages Exacerbate Cisplatin-Induced Mitochondrial Dysfunction and Kidney Injury via miR-195a-5p-SIRT3 Axis, Nature Communications. (2024) 15, no. 1, 10.1038/s41467-024-47842-z, 4383. PMC11116430 38782909

[51] Baisantry A. , Bhayana S. , and Rong S. , et al.Autophagy Induces Prosenescent Changes in Proximal Tubular S3 Segments, Journal of the American Society of Nephrology. (2016) 27, no. 6, 1609–1616, 10.1681/ASN.2014111059, 2-s2.0-84991340473. 26487561 PMC4884098

[52] Livingston M. J. , Ding H.-F. , Huang S. , Hill J. A. , Yin X.-M. , and Dong Z. , Persistent Activation of Autophagy in Kidney Tubular Cells Promotes Renal Interstitial Fibrosis During Unilateral Ureteral Obstruction, Autophagy. (2016) 12, no. 6, 976–998, 10.1080/15548627.2016.1166317, 2-s2.0-84973863166. 27123926 PMC4922446

[53] Wang Y. , Tang C. , and Cai J. , et al.PINK1/Parkin-Mediated Mitophagy Is Activated in Cisplatin Nephrotoxicity to Protect Against Kidney Injury, Cell Death & Disease. (2018) 9, no. 11, 10.1038/s41419-018-1152-2, 2-s2.0-85065555648, 1113. 30385753 PMC6212494

[54] Zhou L. , Zhang L. , and Zhang Y. , et al.PINK1 Deficiency Ameliorates Cisplatin-Induced Acute Kidney Injury in Rats, Frontiers in Physiology. (2019) 10, 10.3389/fphys.2019.01225, 2-s2.0-85073002866, 1225. 31607953 PMC6773839

[55] Wang Z. , Sun W. , Sun X. , Wang Y. , and Zhou M. , Kaempferol Ameliorates Cisplatin Induced Nephrotoxicity by Modulating Oxidative Stress, Inflammation and Apoptosis via ERK and NF-κB Pathways, AMB Express. (2020) 10, no. 1, 10.1186/s13568-020-00993-w, 58. 32219583 PMC7098399

