# Supplementary material for: Yiqi–Wenyang–Tiaoshen Decoction Reduces Cisplatin‐Induced Acute Kidney Injury in Rats Through Autophagy and Apoptosis Signaling Pathways Based on Network Pharmacology and Experimental Validation
Source: Mediators Inflamm. 2026 May 11;2026:5435119. doi: 10.1155/mi/5435119 (PMC13159095; doi:10.1155/mi/5435119)
Supplement: Supplementary file 1 — Supporting Information Table S1: Identified solution compounds of YWT by UPLC‐MS/MS. Table S2: Identified serum compounds of YWT by UPLC‐MS/MS. Table S3: The targets of YWT. Table S4: The targets of cisplatin‐induced AKI. Table S5: The key targets of YWT in cisplatin‐induced AKI. [file MI-2026-5435119-s001.docx]

**Supporting Information**

**Yiqiwen-yangtiao-shen Decoction Reduces** **Cisplatin-Induced Acute Kidney Injury in Rats through Autophagy and Apoptosis Signaling Pathways Based on Network Pharmacology and Experimental Validation**

**Table S1.Identified solution compounds of YWT by UPLC-MS/MS.**

| **No.** | **Rt(Min)** | **Q1(Da)** | **Moluecular Weight (Da)** | **Formula** | **Ionization Model** | **Compounds** | **Class** | **CAS** |
| --- | --- | --- | --- | --- | --- | --- | --- | --- |
| 1. 1 | 4.78 | 267.10 | 268.1039 | C_10_H_13_N_5_O_4_ | [M+H]^+^ | Adenosine | [Purine nucleosides](http://classyfire.wishartlab.com/tax_nodes/C0000479) | 58-61-7 |
|  | 5.88 | 130.03 | 129.0183 | C_5_H_6_O_4_ | [M-H]^-^ | Citraconic acid | [Fatty Acyls](http://classyfire.wishartlab.com/tax_nodes/C0003909) | 498-23-7 |
|  | 2.78 | 347.06 | 348.0704 | C_10_H_14_N_5_O_7_P | [M+H]^+^ | Adenosine 5'-monophosphate | Nucleotides and their derivatives | 61-19-8 |
|  | 8.83 | 216.09 | 217.0972 | C_12_H_12_N_2_O_2_ | [M+H]^+^ | 2,3,4,9-Tetrahydro-1H-β-carboline-3-carboxylic acid | Others | Not Available |
|  | 13.59 | 430.13 | 431.1336 | C_22_H_22_O_9_ | [M+H]^+^ | Ononin | [Isoflavonoids](http://classyfire.wishartlab.com/tax_nodes/C0002506) | 486-62-4 |
|  | 2.27 | 123.03 | 124.0397 | C_6_H_5_NO_2_ | [M+H]^+^ | Nicotinic acid | Others | 59-67-6 |
|  | 22.16 | 280.24 | 279.2332 | C_18_H_32_O_2_ | [M-H]^-^ | Linoleic acid | [Fatty Acyls](http://classyfire.wishartlab.com/tax_nodes/C0003909) | 60-33-3 |
|  | 11.69 | 164.05 | 163.0391 | C_9_H_8_O_3_ | [M-H]^-^ | 2,3-Dihydro-1-benzofuran-2-carboxylic acid | Others | 1914-60-9 |
|  | 2.12 | 109.05 | 110.0606 | C_6_H_7_NO | [M+H]^+^ | 3-Hydroxy-2-methylpyridine | Pyridine Alkaloids | 1121-25-1 |
|  | 16.77 | 284.07 | 285.0755 | C_16_H_12_O_5_ | [M+H]^+^ | Wogonin | Flavonoids | 6665-74-3 |
|  | 1.31 | 119.06 | 120.0659 | C_4_H_9_NO_3_ | [M+H]^+^ | DL-Homoserine | Carboxylic acids and derivatives | 672-15-1 |
|  | 10.14 | 354.10 | 353.0879 | C_16_H_18_O_9_ | [M-H]^-^ | Chlorogenic acid | Phenolic acids | 327-97-9 |
|  | 2.84 | 129.04 | 128.0341 | C_5_H_7_NO_3_ | [M-H]^-^ | 4-Oxoproline | [Carboxylic acids and derivatives](http://classyfire.wishartlab.com/tax_nodes/C0000265) | Not Available |
|  | 6.24 | 132.04 | 131.0339 | C_5_H_8_O_4_ | [M-H]^-^ | Methylsuccinic acid | [Fatty Acyls](http://classyfire.wishartlab.com/tax_nodes/C0003909) | 2174-58-5 |
|  | 1.22 | 155.07 | 156.0769 | C_6_H_9_N_3_O_2_ | [M+H]^+^ | L-Histidine | Amino acids and their derivatives | 71-00-1 |
|  | 16.99 | 254.06 | 255.0651 | C_15_H_10_O_4_ | [M+H]^+^ | Chrysin | Flavonoids | 480-40-0 |
|  | 10.40 | 180.04 | 179.0343 | C_9_H_8_O_4_ | [M-H]^-^ | Caffeic acid | Phenolic acids | 331-39-5 |
|  | 10.07 | 122.04 | 121.0283 | C_7_H_6_O_2_ | [M-H]^-^ | Benzoic acid | [Benzene and substituted derivatives](http://classyfire.wishartlab.com/tax_nodes/C0002279) | 65-85-0 |
|  | 18.01 | 282.15 | 281.1397 | C_15_H_22_O_5_ | [M-H]^-^ | Octyl gallate | [Benzene and substituted derivatives](http://classyfire.wishartlab.com/tax_nodes/C0002279) | 1034-01-1 |
|  | 17.12 | 352.22 | 353.2296 | C_18_H_34_O_5_ | [M+H]^+^ | 9,10,11-trihydroxy-(12z)-12-octadecenoic acid | fatty acid | Not Available |
|  | 10.70 | 173.10 | 172.0971 | C_8_H_15_NO_3_ | [M-H]^-^ | 2-(Acetylamino)hexanoic acid | organic acid | 7682-16-8 |
|  | 5.48 | 134.02 | 152.0569 | C_5_H_5_N_5_O | [M+NH_4_]^+^ | Guanine | Nucleotides and their derivatives | 73-40-5 |
|  | 10.08 | 166.03 | 165.0184 | C_8_H_6_O_4_ | [M-H]^-^ | Isophthalic acid | organic acid | 121-91-5 |
|  | 14.50 | 476.10 | 477.1026 | C_22_H_20_O_12_ | [M+H]^+^ | Hispidulin 7-glucuronide | Flavonoids | 31105-76-7 |
|  | 12.36 | 492.13 | 491.1199 | C_23_H_24_O_12_ | [M-H]^-^ | 2-Hydroxy-3-(5-hydroxy-7,8-dimethoxy-4-oxo-4H-chromen-2-yl)phenyl β-D-glucopyranoside | Terpenoids | Not Available |
|  | 15.89 | 152.12 | 153.1274 | C_10_H_16_O | [M+H]^+^ | (-)-Camphor | [Prenol lipids](http://classyfire.wishartlab.com/tax_nodes/C0000259) | 464-49-3 |
|  | 13.55 | 268.07 | 301.1071 | C_16_H_12_O_4_ | [M+H+MeOH]^+^ | Formononetin | [Isoflavonoids](http://classyfire.wishartlab.com/tax_nodes/C0002506) | 485-72-3 |
|  | 4.79 | 118.03 | 136.0620 | C_5_H_5_N_5_ | [M+NH_4_]^+^ | Adenine | Nucleotides and their derivatives | 73-24-5 |
|  | 11.23 | 336.08 | 337.0919 | C_16_H_18_O_9_ | [M+H]^+^ | (1r,3R,4s,5S)-4-{[(2E)-3-(3,4-dihydroxyphenyl)prop-2-enoyl]oxy}-1,3,5-trihydroxycyclohexane-1-carboxylic acid | Others | Not Available |
|  | 14.50 | 476.10 | 477.1026 | C_22_H_20_O_12_ | [M+H]^+^ | 6-O-Methylscutellarin | [Flavonoids](http://classyfire.wishartlab.com/tax_nodes/C0000334) | 31105-76-7 |
|  | 22.69 | 256.24 | 255.2331 | C_16_H_32_O_2_ | [M-H]^-^ | Palmitic Acid | lipids | 1957-10-3 |
|  | 6.10 | 126.03 | 127.0392 | C_6_H_6_O_3_ | [M+H]^+^ | 5-Hydroxymethyl-2-furaldehyde | [Organooxygen compounds](http://classyfire.wishartlab.com/tax_nodes/C0000323) | 67-47-0 |
|  | 13.01 | 446.08 | 447.0921 | C_21_H_18_O_11_ | [M+H]^+^ | Baicalin | [Flavonoids](http://classyfire.wishartlab.com/tax_nodes/C0000334) | 21967-41-9 |
|  | 5.02 | 251.10 | 252.1094 | C_10_H_13_N_5_O_3_ | [M+H]^+^ | 2'-Deoxyadenosine | Nucleoside | 958-09-8 |
|  | 1.85 | 260.03 | 261.0371 | C_6_H_13_O_9_P | [M+H]^+^ | Glucose 1-phosphate | [Organooxygen compounds](http://classyfire.wishartlab.com/tax_nodes/C0000323) | 59-56-3 |
|  | 5.41 | 170.02 | 169.0134 | C_7_H_6_O_5_ | [M-H]-1 | Gallic acid | Phenolic acids | 149-91-7 |
|  | 1.50 | 137.05 | 138.0550 | C_7_H_7_NO_2_ | [M+H]^+^ | Trigonelline | Alkaloids | 535-83-1 |
|  | 2.88 | 192.03 | 191.0190 | C_6_H_8_O_7_ | [M-H]^-^ | Citric acid | Organic acid | 77-92-9 |
|  | 12.03 | 134.04 | 193.0500 | C_10_H_10_O_4_ | [M-H+HAc]^-^ | Ferulic acid | Phenolic acids | 1135-24-6 |
|  | 14.89 | 254.06 | 255.0648 | C_15_H_10_O_4_ | [M+H]^+^ | Chrysin | [Flavonoids](http://classyfire.wishartlab.com/tax_nodes/C0000334) | 480-40-0 |
|  | 2.44 | 122.05 | 123.0557 | C_6_H_6_N_2_O | [M+H]^+^ | Nicotinamide | Others | 98-92-0 |
|  | 12.66 | 304.06 | 305.0653 | C_15_H_12_O_7_ | [M+H]^+^ | Taxifolin | Flavonoids | 480-18-2 |
|  | 15.52 | 460.10 | 461.1073 | C_22_H_20_O_11_ | [M+H]^+^ | (2S,3S,4S,5R,6S)-3,4,5-trihydroxy-6-[(5-hydroxy-8-methoxy-4-oxo-2-phenyl-4H-chromen-7-yl)oxy]oxane-2-carboxylic acid | Others | Not Available |
|  | 14.01 | 432.11 | 433.1130 | C_21_H_20_O_10_ | [M+H]^+^ | Apigetrin | [Flavonoids](http://classyfire.wishartlab.com/tax_nodes/C0000334) | 578-74-5 |
|  | 15.44 | 470.21 | 471.2199 | C_21_H_36_O_10_ | [M+H]^+^ | (2R,3S,4S,5R,6R)-2-({[(2R,3R,4R)-3,4-dihydroxy-4-(hydroxymethyl)oxolan-2-yl]oxy}methyl)-6-{[(2E)-3,7-dimethylocta-2,6-dien-1-yl]oxy}oxane-3,4,5-triol | Others | Not Available |
|  | 3.73 | 112.03 | 113.0350 | C_4_H_4_N_2_O_2_ | [M+H]^+^ | Uracil | [Diazines](http://classyfire.wishartlab.com/tax_nodes/C0001346) | 66-22-8 |
|  | 15.90 | 136.13 | 137.1325 | C_10_H_18_O | [M+H]^+^ | Eucalyptol | [Oxanes](http://classyfire.wishartlab.com/tax_nodes/C0002012) | 470-82-6 |
|  | 6.75 | 281.11 | 282.1203 | C_11_H_15_N_5_O_4_ | [M+H]^+^ | N6-Me-Adenosine | Purine nucleosides | 60209-41-8 |
|  | 5.79 | 281.15 | 282.1550 | C_11_H_20_O_7_ | [M+H]^+^ | 2-Hydroxy-2-methyl-3-buten-1-yl beta-D-glucopyranoside | carbohydrate derivative | Not Available |
|  | 1.63 | 117.08 | 118.0865 | C_5_H_11_NO_2_ | [M+H]^+^ | Betaine | Alkaloids | 107-43-7 |
|  | 4.64 | 347.06 | 348.0705 | C_10_H_14_N_5_O_7_P | [M+H]^+^ | 3'-Adenosine monophosphate (3'-AMP) | [Ribonucleoside 3'-phosphates](http://classyfire.wishartlab.com/tax_nodes/C0004404) | 84-21-9 |
|  | 1.40 | 382.11 | 365.1052 | C_12_H_22_O_11_ | [M+H-H_2_O]^+^ | D-(+)-Maltose | [Organooxygen compounds](http://classyfire.wishartlab.com/tax_nodes/C0000323) | 69-79-4 |
|  | 19.52 | 296.24 | 295.2280 | C_18_H_34_O_4_ | [M-H]^-^ | (±)9(10)-DiHOME | Fatty Acids | Not Available |
|  | 16.10 | 328.23 | 327.2179 | C_18_H_32_O_5_ | [M-H]^-^ | Corchorifatty acid F | [Fatty Acyls](http://classyfire.wishartlab.com/tax_nodes/C0003909) | 95341-44-9 |
|  | 13.39 | 462.12 | 463.1235 | C_22_H_22_O_11_ | [M+H]^+^ | 5-hydroxy-2-(4-hydroxyphenyl)-6-methoxy-7-{[(2S,3R,4S,5S,6R)-3,4,5-trihydroxy-6-(hydroxymethyl)oxan-2-yl]oxy}-4H-chromen-4-one | Others | Not Available |
|  | 19.45 | 294.22 | 295.2268 | C_18_H_30_O_3_ | [M+H]^+^ | 9-Oxo-10(E),12(E)-octadecadienoic acid | Fatty Acids | 54665-32-6 |
|  | 14.38 | 610.15 | 611.1606 | C_27_H_30_O_16_ | [M+H]^+^ | Rutin | Flavonoids | 153-18-4 |
|  | 13.03 | 224.10 | 207.1015 | C_12_H_16_O_4_ | [M+H-H_2_O]^+^ | Senkyunolide H | Benzofurans | 94596-27-7 |
|  | 12.26 | 548.15 | 549.1597 | C_26_H_28_O_13_ | [M+H]^+^ | 5,7-dihydroxy-2-phenyl-6-[3,4,5-trihydroxy-6-(hydroxymethyl)oxan-2-yl]-8-(3,4,5-trihydroxyoxan-2-yl)-4H-chromen-4-one | Others | Not Available |
|  | 11.71 | 522.21 | 521.2031 | C_26_H_34_O_11_ | [M-H]^-^ | Lariciresinol 4-O-glucoside | Lignans | Not Available |
|  | 1.68 | 129.08 | 130.0864 | C_6_H_11_NO_2_ | [M+H]^+^ | D-(+)-Pipecolinic acid | [Carboxylic acids and derivatives](http://classyfire.wishartlab.com/tax_nodes/C0000265) | 1723-00-8 |
|  | 18.56 | 806.44 | 807.4496 | C_41_H_68_O_14_ | [M+H]^+^ | Astragaloside A | Saponins | 83207-58-3 |
|  | 9.23 | 290.08 | 289.0723 | C_15_H_14_O_6_ | [M-H]^-^ | Catechin | Flavanols | 7295-85-4 |
|  | 1.18 | 132.09 | 131.0815 | C_5_H_12_N_2_O_2_ | [M-H]^-^ | Ornithine | Others | 3184-13-2 |
|  | 9.22 | 359.16 | 360.1650 | C_16_H_22_O_8_ | [M+H]^+^ | Coniferin | Phenolic acids | 531-29-3 |
|  | 11.97 | 284.07 | 285.0754 | C_16_H_12_O_5_ | [M+H]^+^ | (2Z)-6-hydroxy-2-[(4-hydroxy-3-methoxyphenyl)methylidene]-2,3-dihydro-1-benzofuran-3-one | Others | Not Available |
|  | 15.09 | 314.08 | 315.0859 | C_17_H_14_O_6_ | [M+H]^+^ | 5,7-dihydroxy-3,8-dimethoxy-2-phenyl-4H-chromen-4-one | Flavonoids | 33803-42-8 |
|  | 11.69 | 120.06 | 119.0490 | C_8_H_8_O | [M-H]^-^ | Phenylacetaldehyde | Benzene and substituted derivatives | 122-78-1 |
|  | 11.37 | 410.06 | 443.0974 | C_22_H_18_O_10_ | [M+H+MeOH]^+^ | Catechin gallate | Flavonoids | 25615-05-8 |
|  | 18.04 | 234.16 | 235.1693 | C_15_H_22_O_2_ | [M+H]^+^ | 3,5-di-tert-Butyl-4-hydroxybenzaldehyde | Organooxygen compounds | 1620-98-0 |
|  | 1.22 | 103.10 | 104.1074 | C_5_H_13_NO | [M+H]^+^ | Choline | Alkaloids | 62-49-7 |
|  | 1.32 | 147.05 | 148.0605 | C_5_H_9_NO_4_ | [M+H]^+^ | L-Glutamic acid | Amino acids and their derivatives | 56-86-0 |
|  | 12.04 | 174.09 | 173.0811 | C_8_H_14_O_4_ | [M-H]^-^ | Suberic acid | [Fatty Acyls](http://classyfire.wishartlab.com/tax_nodes/C0003909) | 505-48-6 |
|  | 13.35 | 332.18 | 355.1728 | C_16_H_28_O_7_ | [M+Na]^+^ | (2R,3R,4S,5S,6R)-2-{[(2E,6R)-6-hydroxy-2,6-dimethylocta-2,7-dien-1-yl]oxy}-6-(hydroxymethyl)oxane-3,4,5-triol | Others | Not Available |
|  | 5.15 | 329.05 | 330.0600 | C_10_H_12_N_5_O_6_P | [M+H]^+^ | Adenosine 3'5'-cyclic monophosphate | Purine nucleotides | 60-92-4 |
|  | 9.39 | 484.09 | 483.0786 | C_20_H_20_O_14_ | [M-H]^-^ | 1,6-Bis-O-(3,4,5-trihydroxybenzoyl)hexopyranose | Others | Not avaliable |
|  | 12.65 | 450.12 | 449.1090 | C_21_H_22_O_11_ | [M-H]^-^ | Astilbin | Flavonoids | 29838-67-3 |
|  | 21.62 | 255.26 | 256.2635 | C_16_H_33_NO | [M+H]^+^ | Hexadecanamide | Fatty Acyls | 629-54-9 |
|  | 14.32 | 270.05 | 271.0600 | C_15_H_10_O_5_ | [M+H]^+^ | Baicalein | Flavonoids | 491-67-8 |
|  | 16.20 | 374.10 | 375.1071 | C_19_H_18_O_8_ | [M+H]^+^ | 5,2'-Dihydroxy-6,7,8,6'-tetramethoxyflavone | Flavonoids | 55084-08-7 |
|  | 23.09 | 358.31 | 381.2967 | C_21_H_42_O_4_ | [M+Na]^+^ | 1-Stearoylglycerol | Fatty Acyls | 123-94-4 |
|  | 2.84 | 129.04 | 130.0500 | C_5_H_7_NO_3_ | [M+H]^+^ | D-(+)-Pyroglutamic Acid |  | 149-87-1 |
|  | 5.41 | 126.03 | 125.0233 | C_6_H_6_O_3_ | [M-H]^-^ | Pyrogallol | Phenols | 87-66-1 |
|  | 15.16 | 270.05 | 271.0595 | C_15_H_10_O_5_ | [M+H]^+^ | Genistein | Isoflavonoids | 446-72-0 |
|  | 15.37 | 284.07 | 285.0756 | C_16_H_12_O_5_ | [M+H]^+^ | Glycitein | Isoflavonoids | 40957-83-3 |
|  | 11.96 | 446.12 | 447.1284 | C_22_H_22_O_10_ | [M+H]^+^ | 3-(4-hydroxyphenyl)-5-methoxy-7-{[(2S,3R,4S,5S,6R)-3,4,5-trihydroxy-6-(hydroxymethyl)oxan-2-yl]oxy}-4H-chromen-4-one | Others | Not Available |
|  | 8.41 | 354.10 | 353.0880 | C_16_H_18_O_9_ | [M-H]^-^ | Neochlorogenic acid | polyphenolic substance | 906-33-2 |
|  | 13.73 | 736.16 | 717.1469 | C_36_H_30_O_16_ | [M-H-H_2_O]^-^ | (2R)-2-({(2E)-3-[3-{[(1R)-1-Carboxy-2-(3,4-dihydroxyphenyl)ethoxy]carbonyl}-2-(3,4-dihydroxyphenyl)-7-hydroxy-2,3-dihydro-1-benzofuran-4-yl]-2-propenoyl}oxy)-3-(3,4-dihydroxyphenyl)propanoic acid | Others | Not Available |
|  | 12.86 | 367.22 | 350.2174 | C_16_H_28_O_7_ | [M+H-H_2_O]^+^ | (2E)-4-Hydroxy-3,7-dimethyl-2,6-octadien-1-yl beta-D-glucopyranoside | terpene glycoside | 100462-37-1 |
|  | 11.68 | 192.04 | 193.0498 | C_10_H_8_O_4_ | [M+H]^+^ | 7-hydroxy-6-methoxy-2H-chromen-2-one | Coumarins and derivatives | 92-61-5 |
|  | 9.44 | 196.12 | 197.1287 | C_10_H_16_N_2_O_2_ | [M+H]^+^ | 3-(propan-2-yl)-octahydropyrrolo[1,2-a]pyrazine-1,4-dione | Carboxylic acids and derivatives | [27483-18-7](https://commonchemistry.cas.org/detail?cas_rn=27483-18-7) |
|  | 13.52 | 462.08 | 463.0873 | C_21_H_18_O_12_ | [M+H]^+^ | (2S,3S,4S,5R,6S)-6-{[5,7-dihydroxy-2-(4-hydroxyphenyl)-4-oxo-4H-chromen-3-yl]oxy}-3,4,5-trihydroxyoxane-2-carboxylic acid | Others | Not Available |
|  | 11.93 | 148.05 | 147.0441 | C_9_H_8_O_2_ | [M-H]^-^ | trans-Cinnamic acid | Cinnamic acids and derivatives | 140-10-3 |
|  | 16.97 | 194.13 | 195.1380 | C_12_H_18_O_2_ | [M+H]^+^ | Sedanolide | Benzofurans | 6415-59-4 |
|  | 16.55 | 294.22 | 295.2270 | C_18_H_32_O_4_ | [M+H]^+^ | (±)13-HpODE | Fatty Acyls | 23017-93-8 |
|  | 7.53 | 204.09 | 203.0822 | C_11_H_12_N_2_O_2_ | [M-H]^-^ | DL-Tryptophan | Indoles and derivatives | 54-12-6 |
|  | 10.67 | 132.08 | 131.0702 | C_6_H_12_O_3_ | [M-H]^-^ | 6-Hydroxycaproic acid | Hydroxy acids and derivatives | 1191-25-9 |
|  | 1.62 | 132.04 | 191.0553 | C_7_H_12_O_6_ | [M-H+HAc]^-^ | D-(-)-Quinic acid | Organooxygen compounds | 77-95-2 |
|  | 8.94 | 197.09 | 198.0972 | C_6_H_12_O_6_ | [M+H]^+^ | D-(+)-Glucose | Others | 50-99-7 |
|  | 1.37 | 182.08 | 181.0709 | C_6_H_14_O_6_ | [M-H]^-^ | L-Iditol | Organooxygen compounds | 488-45-9 |
|  | 1.60 | 115.03 | 133.0609 | C_4_H_8_N_2_O_3_ | [M+NH_4_]^+^ | Asparagine | Carboxylic acids and derivatives | 70-47-3 |
|  | 1.62 | 150.05 | 195.0503 | C_6_H_12_O_7_ | [M+FA-H]^-^ | Gluconic acid | Others | 526-95-4 |
|  | 11.26 | 433.20 | 434.2024 | C_19_H_28_O_10_ | [M+H]^+^ | (2S,3R,4S,5R)-2-{[(2R,3R,4S,5S,6R)-4,5-dihydroxy-6-(hydroxymethyl)-2-(2-phenylethoxy)oxan-3-yl]oxy}oxane-3,4,5-triol | Others | Not Available |
|  | 22.42 | 328.19 | 327.1816 | C_17_H_28_O_6_ | [M-H]^-^ | (-)-Spiculisporic acid | organooxygen compound | 469-77-2 |
|  | 10.69 | 168.04 | 167.0341 | C_8_H_8_O_4_ | [M-H]^-^ | 3,4-Dihydroxyphenylacetic acid | Phenols | 102-32-9 |
|  | 6.67 | 242.09 | 241.0830 | C_10_H_14_N_2_O_5_ | [M-H]^-^ | Thymidine | Nucleotides and their derivatives | 50-89-5 |
|  | 14.15 | 300.06 | 299.0565 | C_16_H_12_O_6_ | [M-H]^-^ | Hispidulin | Flavonoids | 1447-88-7 |
|  | 1.22 | 174.11 | 175.1190 | C_6_H_14_N_4_O_2_ | [M+H]^+^ | DL-Arginine | Carboxylic acids and derivatives | 7200-25-1 |
|  | 13.94 | 274.08 | 257.0809 | C_15_H_12_O_4_ | [M+H-H_2_O]^+^ | (3R)-8-hydroxy-3-(4-hydroxyphenyl)-3,4-dihydro-1H-2-benzopyran-1-one | organic heterocyclic compound | Not available |
|  | 1.40 | 542.12 | 543.1320 | C_25_H_28_O_11_ | [M+H]^+^ | (1S,4aS,7aS)-7-({[(2E)-3-phenylprop-2-enoyl]oxy}methyl)-1-{[(2S,3R,4S,5S,6R)-3,4,5-trihydroxy-6-(hydroxymethyl)oxan-2-yl]oxy}-1H,4aH,5H,7aH-cyclopenta[c]pyran-4-carboxylic acid | Others | Not Available |
|  | 14.89 | 416.11 | 417.1175 | C_21_H_20_O_9_ | [M+H]^+^ | Daidzin | Isoflavonoids | 552-66-9 |
|  | 17.11 | 312.23 | 295.2268 | C_18_H_30_O_3_ | [M+H-H_2_O]^+^ | 13(S)-HOTrE | Fatty Acyls | 87984-82-5 |
|  | 15.699 | 166.06 | 167.0703 | C_9_ H_10_ O_3_ | [M+H]^+^ | Apocynin | Phenolic compound | 498-02-2 |
|  | 5.04 | 174.02 | 173.0085 | C_6_H_6_O_6_ | [M-H]^-^ | 1,2,3-cyclopropanetricarboxylic acid | Carboxylic acids and derivatives | 48126-70-1 |
|  | 12.51 | 178.03 | 179.0339 | C_9_H_6_O_4_ | [M+H]^+^ | Aesculetin | Coumarins and derivatives | 305-01-1 |
|  | 10.06 | 208.04 | 209.0445 | C_10_H_8_O_5_ | [M+H]^+^ | Fraxetin | Coumarins and derivatives | 574-84-5 |
|  | 21.65 | 304.24 | 303.2334 | C_20_H_32_O_2_ | [M-H]^-^ | Arachidonic acid | [Fatty Acyls](http://classyfire.wishartlab.com/tax_nodes/C0003909) | 506-32-1 |
|  | 21.15 | 178.06 | 161.0598 | C_10_H_10_O_3_ | [M+H-H_2_O]^+^ | 4-Methoxycinnamic acid | Cinnamic acids and derivatives | 943-89-5 |
|  | 11.69 | 564.15 | 565.1554 | C_26_H_28_O_14_ | [M+H]^+^ | Schaftoside | Flavonoids | 51938-32-0 |
|  | 7.49 | 154.03 | 153.0184 | C_7_H_6_O_4_ | [M-H]^-^ | Gentisic acid | Phenolic acids | 490-79-9 |
|  | 9.10 | 328.12 | 327.1089 | C_15_H_20_O_8_ | [M-H]^-^ | 4-Acetyl-3-hydroxy-5-methylphenyl β-D-glucopyranoside | Others | Not available |
|  | 12.651 | 286.05 | 285.04065 | C_15_ H_10_ O_6_ | [M-H]^-^ | Luteolin | [Flavonoids](http://classyfire.wishartlab.com/tax_nodes/C0000334) | 491-70-3 |
|  | 10.64 | 304.06 | 305.0657 | C_15_H_12_O_7_ | [M+H]^+^ | (2R,3R)-2-(2,6-Dihydroxyphenyl)-3,5,7-trihydroxy-2,3-dihydro-4H-chromen-4-one | Others | Not Available |
|  | 7.52 | 187.06 | 188.0705 | C_11_H_9_NO_2_ | [M+H]^+^ | trans-3-Indoleacrylic acid | Others | Not Available |
|  | 11.64 | 311.12 | 312.1230 | C_19_H_13_N_5_ | [M+H]^+^ | 2-[6-(1H-benzo[d]imidazol-2-yl)-2-pyridyl]-1H-benzo[d]imidazole | Others | Not Available |
|  | 16.11 | 292.20 | 275.2007 | C_18_H_28_O_3_ | [M+H-H_2_O]^+^ | 12-oxo Phytodienoic Acid | Fatty Acyls | 85551-10-6 |
|  | 1.66 | 362.06 | 363.0685 | C_17_H_16_N_4_S_2_ | [M+H]^+^ | 4-ethyl-5-(10H-phenothiazin-10-ylmethyl)-2,4-dihydro-3H-1,2,4-triazole-3-thione | Others | Not available |
|  | 13.81 | 302.04 | 303.0501 | C_15_H_10_O_7_ | [M+H]^+^ | 2-(2,4-dihydroxyphenyl)-3,5,7-trihydroxy-4H-chromen-4-one | Flavonoids | 480-16-0 |
|  | 1.19 | 261.06 | 262.0688 | C_13_H_11_NO_5_ | [M+H]^+^ | Oxolinic acid | Quinolines and derivatives | Not Available |
|  | 21.47 | 354.28 | 337.2738 | C_21_H_38_O_4_ | [M+H-H_2_O]^+^ | 1-Linoleoyl glycerol | Fatty Acyls | 2277-28-3 |
|  | 10.12 | 152.12 | 153.1275 | C_10_H_16_O | [M+H]^+^ | D-(+)-Camphor | Prenol lipids | Not Available |
|  | 7.02 | 167.06 | 168.0658 | C_8_H_9_NO_3_ | [M+H]^+^ | Pyridoxal | Pyridines and derivatives | 66-72-8 |
|  | 1.77 | 192.03 | 191.0190 | C_6_H_8_O_7_ | [M-H]^-^ | Isocitric acid | Carboxylic acids and derivatives | 320-77-4 |
|  | 14.22 | 188.10 | 187.0969 | C_9_H_16_O_4_ | [M-H]^-^ | Azelaic acid | Fatty Acyls | 123-99-9 |
|  | 14.28 | 594.16 | 593.1516 | C_27_H_30_O_15_ | [M-H]^-^ | 2-(3,4-Dihydroxyphenyl)-5-hydroxy-4-oxo-4H-chromen-7-yl 6-O-(6-deoxy-alpha-L-mannopyranosyl)-beta-D-glucopyranoside | Others | Not Available |
|  | 10.40 | 340.13 | 341.1385 | C_20_H_22_O_6_ | [M+H]^+^ | 4-[(1S,3aR,4S,6aR)-4-(4-hydroxy-3-methoxyphenyl)-hexahydrofuro[3,4-c]furan-1-yl]-2-methoxyphenol | Others | Not Available |
|  | 11.94 | 246.10 | 245.0932 | C_13_H_14_N_2_O_3_ | [M-H]^-^ | 2-(acetylamino)-3-(1H-indol-3-yl)propanoic acid | Amino Acids and Derivatives | 87-32-1 |
|  | 6.15 | 281.11 | 282.1199 | C_1_H_15_N_5_O_4_ | [M+H]^+^ | 2'-O-Methyladenosine | Purine nucleosides | 2140-79-6 |
|  | 12.72 | 522.14 | 523.1446 | C_24_H_26_O_13_ | [M+H]^+^ | 5-hydroxy-3-(5-hydroxy-2,4-dimethoxyphenyl)-6-methoxy-7-{[3,4,5-trihydroxy-6-(hydroxymethyl)oxan-2-yl]oxy}-4H-chromen-4-one | Others | Not Available |
|  | 11.02 | 786.26 | 785.2521 | C_35_H_46_O_20_ | [M-H]^-^ | Phlinoside A | Others | Not Available |
|  | 13.85 | 288.06 | 287.0566 | C_15_H_12_O_6_ | [M-H]^-^ | 2-(3,4-dihydroxyphenyl)-5,7-dihydroxy-3,4-dihydro-2H-1-benzopyran-4-one | Flavonoids | 4049-38-1 |
|  | 19.52 | 278.22 | 279.2319 | C_18_H_30_O_2_ | [M+H]^+^ | α-Eleostearic acid | [Fatty Acyls](http://classyfire.wishartlab.com/tax_nodes/C0003909) | Not Available |
|  | 16.03 | 268.07 | 269.0807 | C_16_H_12_O_4_ | [M+H]^+^ | 7-hydroxy-3-(4-methoxyphenyl)-4H-chromen-4-one | Isoflavonoids | [485-72-3](https://commonchemistry.cas.org/detail?cas_rn=485-72-3) |
|  | 13.33 | 236.07 | 237.0753 | C_12_H_12_O_5_ | [M+H]^+^ | 6,7,8-trimethoxy-2H-chromen-2-one | Heterocyclic Compounds | 6035-49-0 |
|  | 22.94 | 255.26 | 256.2635 | C_16_H_33_NO | [M+H]^+^ | N,N-Diethyldodecanamide | Amides | 3352-87-2 |
|  | 16.53 | 330.24 | 329.2335 | C_18_H_34_O_5_ | [M-H]^-^ | (15Z)-9,12,13-Trihydroxy-15-octadecenoic acid | Fatty Acyls | Not Available |
|  | 17.06 | 310.21 | 311.2216 | C_18_H_32_O_5_ | [M+H]^+^ | (11E,15Z)-9,10,13-trihydroxyoctadeca-11,15-dienoic acid | Fatty Acyls | 185148-53-2 |
|  | 14.61 | 446.12 | 447.1255 | C_22_H_22_O_10_ | [M+H]^+^ | 3-(4-hydroxyphenyl)-7-methoxy-5-{[(3R,4S,5S,6R)-3,4,5-trihydroxy-6-(hydroxymethyl)oxan-2-yl]oxy}-4H-chromen-4-one | Others | Not Available |
|  | 8.65 | 273.10 | 274.1074 | C_15_H_17_NO_5_ | [M+H]^+^ | Obscurolide A1 | amino acid | 144397-99-9 |
|  | 12.58 | 193.11 | 194.1179 | C_11_H_15_NO_2_ | [M+H]^+^ | 2-oxa-4-azatetracyclo[6.3.1.1~6,10~.0~1,5~]tridecan-3-one | Others | Not Available |
|  | 17.30 | 148.09 | 149.0962 | C_10_H_12_O | [M+H]^+^ | Cuminaldehyde | [Prenol lipids](http://classyfire.wishartlab.com/tax_nodes/C0000259) | 122-03-2 |
|  | 12.28 | 214.08 | 473.1665 | C_20_H_28_O_10_ | [2M+FA-H]^-^ | (2E)-3-Phenyl-2-propen-1-yl 6-O-beta-D-arabinofuranosyl-beta-D-glucopyranoside | Others | Not Available |
|  | 13.03 | 246.09 | 247.0940 | C_14_H_14_O_4_ | [M+H]^+^ | (2S)-2-(2-hydroxypropan-2-yl)-2H,3H,7H-furo[3,2-g]chromen-7-one | Coumarins and derivatives | 13849-08-6 |
|  | 9.46 | 432.16 | 431.1560 | C_19_H_28_O_11_ | [M-H]^-^ | 2-(4-Hydroxyphenyl)ethyl6-O-[(2R,3R,4R)-3,4-dihydroxy-4-(hydroxymethyl)tetrahydro-2-furanyl]-beta-D-glucopyranoside | Others | Not Available |
|  | 8.41 | 151.06 | 152.0708 | C_8_H_9_NO_2_ | [M+H]^+^ | Ethyl nicotinate | Pyridines and derivatives | Not Available |
|  | 12.31 | 164.05 | 147.0440 | C_9_H_8_O_3_ | [M+H-H_2_O]^+^ | 2-Hydroxycinnamic acid | Cinnamic acids and derivatives | 614-60-8 |
|  | 6.09 | 140.05 | 141.0547 | C_7_H_8_O_3_ | [M+H]^+^ | 2-Methoxyresorcinol | Phenols | 29267-67-2 |
|  | 2.08 | 521.20 | 522.2033 | C_18_H_32_O_16_ | [M+H]^+^ | D-Raffinose | Organooxygen compounds | 512-69-6 |
|  | 7.93 | 374.12 | 373.1143 | C_16_H_22_O_10_ | [M-H]^-^ | Geniposidic acid | Prenol lipids | 27741-01-1 |
|  | 7.52 | 114.05 | 132.0809 | C_9_H_9_N | [M+NH_4_]^+^ | 6-Methylindole | Indoles and derivatives | 3420-02-8 |
|  | 13.76 | 193.11 | 194.1178 | C_11_H_15_NO_2_ | [M+H]^+^ | Butyl 4-aminobenzoate | Benzene and substituted derivatives | Not Available |
|  | 10.94 | 448.10 | 449.1083 | C_21_H_20_O_11_ | [M+H]^+^ | 4-(3,4-dihydroxyphenyl)-7-hydroxy-5-{[(2S,3R,4S,5S,6R)-3,4,5-trihydroxy-6-(hydroxymethyl)oxan-2-yl]oxy}-2H-chromen-2-one | Others | Not Available |
|  | 8.60 | 147.07 | 148.0757 | C_9_H_9_NO | [M+H]^+^ | 5-Methoxyindole | Indoles and derivatives | Not Available |
|  | 15.13 | 178.10 | 179.1066 | C_11_H_14_O_2_ | [M+H]^+^ | 4-Isobutylbenzoic acid | Others | 38861-88-0 |
|  | 11.95 | 478.11 | 477.1044 | C_22_H_22_O_12_ | [M-H]^-^ | 6-O-[(2E)-3-(4-Hydroxyphenyl)-2-propenoyl]-1-O-(3,4,5-trihydroxybenzoyl)hexopyranose | Others | Not Available |
|  | 2.39 | 228.15 | 229.1549 | C_11_H_20_N_2_O_3_ | [M+H]^+^ | Prolylleucine | Carboxylic acids and derivatives | Not Available |
|  | 2.90 | 112.01 | 111.0074 | C_5_H_4_O_3_ | [M-H]^-^ | 2-Furoic acid | Furans | 88-14-2 |
|  | 10.62 | 239.09 | 240.1019 | C_15_H_13_NO_2_ | [M+H]^+^ | 4,4'-dimethoxy[1,1'-biphenyl]-2-carbonitrile |  |  |
|  | 3.12 | 164.05 | 182.0814 | C_9_H_10_O_4_ | [M+NH_4_]^+^ | 3-(3,4-dihydroxyphenyl)propanoic acid | Phenylpropanoic acids | 23028-17-3 |
|  | 18.53 | 296.24 | 297.2425 | C_18_H_34_O_4_ | [M+H]^+^ | (+/-)12(13)-DiHOME | Fatty Acyls | Not Available |
|  | 9.16 | 432.16 | 431.1559 | C_19_H_28_O_11_ | [M-H]^-^ | 2-(4-Hydroxyphenyl)ethyl 6-O-[(2R,3R,4R)-3,4-dihydroxy-4-(hydroxymethyl)tetrahydro-2-furanyl]-beta-D-glucopyranoside | Others | Not Available |
|  | 4.48 | 288.13 | 289.1397 | C_15_H_22_O_4_ | [M+H]^+^ | 1,9b-Dihydroxy-6,6,9a-trimethyl-5,5a,6,7,8,9,9a,9b-octahydronaphtho[1,2-c]furan-3(1H)-one | Others | Not Available |
|  | 12.04 | 162.03 | 195.0653 | C_9_H_6_O_3_ | [M+H+MeOH]^+^ | 7-Hydroxycoumarine | Coumarins and derivatives | 93-35-6 |
|  | 8.41 | 462.17 | 461.1670 | C_20_H_30_O_12_ | [M-H]^-^ | Bioside | Steroids and steroid derivatives | Not Available |
|  | 10.48 | 138.03 | 139.0392 | C_7_H_6_O_3_ | [M+H]^+^ | 3,4-Dihydroxybenzaldehyde | Organooxygen compounds | 139-85-5 |
|  | 11.91 | 307.11 | 308.1131 | C_13_H_17_N_5_O_2_S | [M+H]^+^ | 1-[(1-methyl-1H-imidazol-4-yl)sulfonyl]-4-(2-pyridinyl)piperazine | Others | Not Available |
|  | 11.68 | 330.17 | 331.1728 | C_17_H_22_N_4_O_3_ | [M+H]^+^ | Ethyl3-cyclopropyl-1-methyl-4-morpholino-1H-pyrazolo[3,4-b]pyridine-5-carboxylate | Others | Not Available |
|  | 3.74 | 244.07 | 243.0623 | C_9_H_12_N_2_O_6_ | [M-H]^-^ | Uridine | Nucleotides and their derivatives | 58-96-8 |
|  | 13.65 | 338.10 | 339.1074 | C_16_H_18_O_8_ | [M+H]^+^ | 4-Methylumbelliferyl-α-D-glucopyranoside | Others | Not Available |
|  | 14.69 | 240.04 | 241.0490 | C_14_H_8_O_4_ | [M+H]^+^ | Alizarin | Anthracenes | Not Available |
|  | 18.09 | 312.23 | 311.2232 | C_18_H_32_O_4_ | [M-H]^-^ | (±)9-HpODE | Fatty Acyls | Not Available |
|  | 13.45 | 288.06 | 289.0706 | C_15_H_12_O_6_ | [M+H]^+^ | Eriodictyol | Flavonoids | 552-58-9 |

**Table S2. Identified serum compounds of YWT by UPLC-MS/MS.**

| **No.** | **Rt(Min)** | **Q1(Da)** | **Moluecular Weight (Da)** | **Formula** | **Ionization Model** | **Compounds** | **Class** | **CAS** |
| --- | --- | --- | --- | --- | --- | --- | --- | --- |
| 1. 1 | 23.82 | 284.3 | 283.2653 | C_18_H_36_O_2_ | [M-H]^-^ | Stearic acid | Lignans and Coumarins | 1957-11-4 |
|  | 5.56 | 165.1 | 166.0868 | C_9_H_11_NO_2_ | [M+H]^+^ | L-Phenylalanine | amino acids and derivatives | 63-91-2 |
|  | 22.77 | 282.3 | 281.2494 | C_18_H_34_O_2_ | [M-H]^-^ | Oleic Acid | [Fatty Acyls](http://classyfire.wishartlab.com/tax_nodes/C0003909) | 112-80-1 |
|  | 1.54 | 111.0 | 112.0513 | C_4_H_5_N_3_O | [M+H]^+^ | Cytosine | Nucleotides and their derivatives | 71-30-7 |
|  | 2.69 | 131.1 | 132.1024 | C_6_H_13_NO_2_ | [M+H]^+^ | L-Isoleucine | Amino acids and their derivatives | 73-32-5 |
|  | 22.01 | 280.2 | 279.2337 | C_18_H_32_O_2_ | [M-H]^-^ | Linoleic acid | [Fatty Acyls](http://classyfire.wishartlab.com/tax_nodes/C0003909) | 60-33-3 |
|  | 7.12 | 285.1 | 286.1044 | C_11_H_15_N_3_O_6_ | [M+H]^+^ | N4-Acetylcytidine | [Pyrimidine nucleosides](http://classyfire.wishartlab.com/tax_nodes/C0000480) | 3768-18-1 |
|  | 7.47 | 204.1 | 188.0711 | C_11_H_12_N_2_O_2_ | [M+H-NH_3_]^+^ | DL-Tryptophan | Indoles and derivatives | 54-12-6 |
|  | 1.39 | 131.1 | 132.0772 | C_4_H_9_N_3_O_2_ | [M+H]^+^ | Creatine | [Carboxylic acids and derivatives](http://classyfire.wishartlab.com/tax_nodes/C0000265) | 57-00-1 |
|  | 17.72 | 399.3 | 400.3429 | C_23_H_45_NO_4_ | [M+H]^+^ | Palmitoylcarnitine | [Fatty Acyls](http://classyfire.wishartlab.com/tax_nodes/C0003909) | 2364-67-2 |
|  | 18.63 | 430.3 | 431.2776 | C_24_H_40_O_5_ | [M+H]^+^ | 4-{[5-(7-hydroxy-5,5,8a-trimethyl-2-methylidene-decahydronaphthalen-1-yl)-3-methylpentyl]oxy}-4-oxobutanoic acid | Others | Not Available |
|  | 1.72 | 149.1 | 150.0588 | C_5_H_11_NO_2_S | [M+H]^+^ | L-(-)-Methionine | Carboxylic acids and derivatives | 63-68-3 |
|  | 14.46 | 446.1 | 447.0935 | C_21_H_18_O_11_ | [M+H]^+^ | Baicalin | [Flavonoids](http://classyfire.wishartlab.com/tax_nodes/C0000334) | 21967-41-9 |
|  | 22.54 | 256.2 | 255.2334 | C_16_H_32_O_2_ | [M-H]^-^ | Palmitic Acid | lipids | 1957-10-3 |
|  | 1.59 | 203.1 | 204.1235 | C_9_H_17_NO_4_ | [M+H]^+^ | Acetyl-L-carnitine | Fatty Acyls | 3040-38-8 |
|  | 9.12 | 179.1 | 180.0661 | C_9_H_9_NO_3_ | [M+H]^+^ | Hippuric acid | [Benzene and substituted derivatives](http://classyfire.wishartlab.com/tax_nodes/C0002279) | 495-69-2 |
|  | 1.57 | 129.0 | 147.0768 | C_5_H_10_N_2_O_3_ | [M+NH_4_]^+^ | DL-Glutamine | α-amino acid | 6899-04-3 |
|  | 12.57 | 298.1 | 321.1319 | C_15_H_22_O_6_ | [M+Na]^+^ | methyl2-(1,3,5-trihydroxy-4a-methyl-8-oxo-decahydronaphthalen-2-yl)prop-2-enoate | Others | Not Available |
|  | 23.33 | 584.3 | 585.2716 | C_33_H_36_N_4_O_6_ | [M+H]^+^ | Bilirubin | [Tetrapyrroles and derivatives](http://classyfire.wishartlab.com/tax_nodes/C0001455) | 635-65-4 |
|  | 1.41 | 161.1 | 162.1130 | C_7_H_15_NO_3_ | [M+H]^+^ | DL-Carnitine | Fatty Acyls | 406-76-8 |
|  | 9.79 | 193.1 | 194.0819 | C_10_H_11_NO_3_ | [M+H]^+^ | Phenylacetylglycine | Carboxylic acids and derivatives | 500-98-1 |
|  | 1.37 | 113.1 | 136.0486 | C_4_H_7_N_3_O | [M+Na]^+^ | Creatinine | Carboxylic acids and derivatives | 60-27-5 |
|  | 1.71 | 185.1 | 218.1392 | C_10_H_19_NO_4_ | [M+H+MeOH]^+^ | Propionylcarnitine | Fatty Acyls | 20064-19-1 |
|  | 1.63 | 143.1 | 144.1023 | C_7_H_13_NO_2_ | [M+H]^+^ | DL-Stachydrine | Carboxylic acids and derivatives | 471-87-4 |
|  | 15.77 | 346.2 | 347.2224 | C_21_H_30_O_4_ | [M+H]^+^ | Corticosterone | [Steroids and steroid derivatives](http://classyfire.wishartlab.com/tax_nodes/C0000258) | 50-22-6 |
|  | 20.13 | 379.2 | 380.2565 | C_18_H_38_NO_5_P | [M+H]^+^ | D-Erythro-sphingosine 1-phosphate | Others | Not Available |
|  | 14.01 | 432.11 | 433.1130 | C_21_H_20_O_10_ | [M+H]^+^ | Apigetrin | [Flavonoids](http://classyfire.wishartlab.com/tax_nodes/C0000334) | 578-74-5 |
|  | 22.13 | 304.2 | 305.2477 | C_20_H_32_O_2_ | [M+H]^+^ | Arachidonic acid | Lignans and Coumarins | 506-32-1 |
|  | 5.41 | 170.02 | 169.0134 | C_7_H_6_O_5_ | [M-H]-1 | Gallic acid | Phenolic acids | 149-91-7 |
|  | 9.37 | 259.2 | 260.1866 | C_13_H_25_NO_4_ | [M+H]^+^ | Hexanoylcarnitine | Fatty Acyls | 6418-78-6 |
|  | 15.25 | 460.1 | 461.1088 | C_22_H_20_O_11_ | [M+H]^+^ | (2S,3S,4S,5R,6S)-3,4,5-trihydroxy-6-[(5-hydroxy-8-methoxy-4-oxo-2-phenyl-4H-chromen-7-yl)oxy]oxane-2-carboxylic acid | Flavonoids | 31105-76-7 |
|  | 17.57 | 454.3 | 453.2873 | C_24_H_40_O_5_ | [M-H]^-^ | β-Muricholic acid | Steroids and steroid derivatives | 2393-59-1 |
|  | 10.79 | 133.1 | 134.0607 | C_8_H_7_NO | [M+H]^+^ | 2-Oxindole | Indoles and derivatives | 59-48-3 |
|  | 19.91 | 392.3 | 391.2865 | C_24_H_40_O_4_ | [M-H]^-^ | Deoxycholic acid | Steroids and steroid derivatives | 83-44-3 |
|  | 12.03 | 134.04 | 193.0500 | C_10_H_10_O_4_ | [M-H+HAc]^-^ | Ferulic acid | Phenolic acids | 1135-24-6 |
|  | 22.43 | 306.3 | 307.2638 | C_20_H_34_O_2_ | [M+H]^+^ | 8Z,11Z,14Z-Eicosatrienoic acid | Fatty Acyls | 1783-84-2 |
|  | 2.40 | 122.0 | 123.0560 | C_6_H_6_N_2_O | [M+H]^+^ | Nicotinamide | Others | 98-92-0 |
|  | 19.62 | 326.2 | 327.2318 | C_22_H_32_O_3_ | [M+H]^+^ | 14(S)-HDHA | Fatty Acyls | 119433-37-3 |
|  | 21.84 | 328.2 | 327.2336 | C_22_H_32_O_2_ | [M-H]^-^ | Docosahexaenoic Acid | Fatty Acids | 6217-54-5 |
|  | 1.77 | 260.0 | 259.0230 | C_6_H_13_O_9_P | [M-H]^-^ | D-Glucose 6-phosphate | Others | 56-73-5 |
|  | 3.21 | 168.0 | 169.0362 | C_5_H_4_N_4_O_3_ | [M+H]^+^ | Uric acid | Imidazopyrimidines | 69-93-2 |
|  | 15.08 | 238.1 | 261.1105 | C_16_H_12_N_4_ | [M+Na]^+^ | 4-phenylbenzo[4,5]imidazo[1,2-a]pyrimidin-2-amine | Pyrimidines | Not available |
|  | 16.71 | 299.3 | 300.2904 | C_18_H_37_NO_2_ | [M+H]^+^ | D-Sphingosine | Organonitrogen compounds | 123-78-4 |
|  | 17.82 | 234.2 | 235.1696 | C_15_H_22_O_2_ | [M+H]^+^ | 3,5-di-tert-Butyl-4-hydroxybenzaldehyde | Organooxygen compounds | 1620-98-0 |
|  | 16.11 | 249.2 | 282.2046 | C_15_H_23_NO_2_ | [M+H+MeOH]^+^ | N-Desmethyltramadol | [Phenol ethers](http://classyfire.wishartlab.com/tax_nodes/C0002341) | 75377-45-6 |
|  | 16.99 | 254.06 | 255.0651 | C_15_H_10_O_4_ | [M+H]^+^ | Chrysin | Flavonoids | 480-40-0 |
|  | 2.90 | 129.0 | 128.0345 | C_5_H_7_NO_3_ | [M-H]^-^ | 4-Oxoproline | [Carboxylic acids and derivatives](http://classyfire.wishartlab.com/tax_nodes/C0000265) | Not Available |
|  | 7.83 | 157.1 | 156.0660 | C_7_H_11_NO_3_ | [M-H]^-^ | N-Tigloylglycine | [Carboxylic acids and derivatives](http://classyfire.wishartlab.com/tax_nodes/C0000265) | 35842-45-6 |
|  | 1.61 | 125.0 | 126.0225 | C_2_H_7_NO_3_S | [M+H]^+^ | Taurine | [Organic sulfonic acids and derivatives](http://classyfire.wishartlab.com/tax_nodes/C0004434) | 107-35-7 |
|  | 22.90 | 206.1 | 413.2669 | C_26_H_36_O_4_ | [2M+H]^+^ | 5-[(10Z)-14-(3,5-dihydroxyphenyl)tetradec-10-en-1-yl]benzene-1,3-diol | benzenediols | Not Available |
|  | 14.50 | 476.10 | 477.1026 | C_22_H_20_O_12_ | [M+H]^+^ | 6-O-Methylscutellarin | [Flavonoids](http://classyfire.wishartlab.com/tax_nodes/C0000334) | 31105-76-7 |
|  | 19.04 | 300.2 | 301.2168 | C_20_H_30_O_3_ | [M+H]^+^ | (±)8-HEPE | Fatty Acyls | 99217-77-3 |
|  | 9.96 | 160.1 | 159.0657 | C_7_H_12_O_4_ | [M-H]^-^ | Pimelic acid | [Fatty Acyls](http://classyfire.wishartlab.com/tax_nodes/C0003909) | 111-16-0 |
|  | 1.32 | 119.1 | 120.0661 | C_4_H_9_NO_3_ | [M+H]^+^ | DL-Homoserine | Amino Acids | 1927-25-9 |
|  | 10.70 | 189.0 | 190.0505 | C_10_H_7_NO_3_ | [M+H]^+^ | α-Cyano-3-hydroxycinnamic acid | Cinnamic acids and derivatives | Not Available |
|  | 22.46 | 330.3 | 329.2495 | C_22_H_34_O_2_ | [M-H]^-^ | Docosapentaenoic acid | Fatty Acyls | 24880-45-3 |
|  | 21.62 | 255.26 | 256.2635 | C_16_H_33_NO | [M+H]^+^ | Hexadecanamide | Fatty Acyls | 629-54-9 |
|  | 19.64 | 302.2 | 303.2317 | C_20_H_32_O_3_ | [M+H]^+^ | (±)11(12)-EET | Fatty Acyls | 200960-01-6 |
|  | 8.41 | 354.10 | 353.0880 | C_16_H_18_O_9_ | [M-H]^-^ | Neochlorogenic acid | polyphenolic substance | 906-33-2 |
|  | 13.37 | 189.1 | 190.0871 | C_11_H_11_NO_2_ | [M+H]^+^ | Methyl indole-3-acetate | Indoles and derivatives | 1912-33-0 |
|  | 8.52 | 317.1 | 318.1558 | C_14_H_20_O_7_ | [M+H]^+^ | (2R,3S,4S,5R,6R)-2-(hydroxymethyl)-6-[2-(4-hydroxyphenyl)ethoxy]oxane-3,4,5-triol | Organooxygen compounds | 10338-51-9 |
|  | 1.21 | 174.1 | 175.1194 | C_6_H_14_N_4_O_2_ | [M+H]^+^ | DL-Arginine | Carboxylic acids and derivatives | 7200-25-1 |
|  | 20.53 | 330.3 | 331.2634 | C_22_H_34_O_2_ | [M+H]^+^ | all-cis-4,7,10,13,16-Docosapentaenoic acid | Fatty Acyls | 25182-74-5 |
|  | 11.79 | 174.1 | 173.0816 | C_8_H_14_O_4_ | [M-H]^-^ | Suberic acid | [Fatty Acyls](http://classyfire.wishartlab.com/tax_nodes/C0003909) | 505-48-6 |
|  | 10.66 | 205.0 | 206.0457 | C_10_H_7_NO_4_ | [M+H]^+^ | Xanthurenic acid | [Quinolines and derivatives](http://classyfire.wishartlab.com/tax_nodes/C0001253) | 59-00-7 |
|  | 22.02 | 262.2 | 263.2372 | C_18_H_32_O_2_ | [M+H]^+^ | octadec-9-ynoic acid | Fatty Acyls | 506-24-1 |
|  | 18.73 | 318.2 | 319.2275 | C_20_H_32_O_4_ | [M+H]^+^ | (+/-)12-HpETE | Fatty Acyls | 71030-35-8 |
|  | 19.30 | 294.2 | 277.2168 | C_18_H_32_O_4_ | [M+H-H_2_O]^+^ | (±)13-HpODE | Fatty Acyls | 23017-93-8 |
|  | 7.47 | 145.1 | 146.0605 | C_9_H_7_NO | [M+H]^+^ | 4-Indolecarbaldehyde | Indoles and derivatives | 1074-86-8 |
|  | 1.66 | 197.1 | 198.0978 | C_6_H_12_O_6_ | [M+H]^+^ | D-(+)-Glucose | Others | 50-99-7 |
|  | 11.20 | 166.1 | 165.0553 | C_9_H_10_O_3_ | [M-H]-1 | L-(-)-3-Phenyllactic acid | [Phenylpropanoic acids](http://classyfire.wishartlab.com/tax_nodes/C0002551) | 20312-36-1 |
|  | 15.52 | 157.1 | 158.1544 | C_9_H_19_NO | [M+H]^+^ | 2,2,6,6-Tetramethyl-1-piperidinol (TEMPO) | Piperidines | Not Available |
|  | 10.40 | 180.04 | 179.0343 | C_9_H_8_O_4_ | [M-H]^-^ | Caffeic acid | Phenolic acids | 331-39-5 |
|  | 1.62 | 228.1 | 229.1552 | C_11_H_20_N_2_O_3_ | [M+H]^+^ | Prolylleucine | Carboxylic acids and derivatives | Not Available |
|  | 11.93 | 148.05 | 147.0441 | C_9_H_8_O_2_ | [M-H]^-^ | trans-Cinnamic acid | Cinnamic acids and derivatives | 140-10-3 |
|  | 1.42 | 196.1 | 195.0510 | C_6_H_12_O_7_ | [M-H]^-^ | Gluconic acid | Others | 526-95-4 |
|  | 8.94 | 159.0 | 192.0663 | C_10_H_9_NO_3_ | [M+H+MeOH]^+^ | 5-Hydroxyindole-3-acetic acid | Indoles and derivatives | 54-16-0 |
|  | 16.48 | 230.2 | 229.1447 | C_12_H_22_O_4_ | [M-H]^-^ | Dodecanedioic acid | [Fatty Acyls](http://classyfire.wishartlab.com/tax_nodes/C0003909) | 693-23-2 |
|  | 19.64 | 342.2 | 343.2243 | C_20_H_32_O_3_ | [M+H]^+^ | 5-(1,2,4a,5-tetramethyl-7-oxo-1,2,3,4,4a,7,8,8a-octahydronaphthalen-1-yl)-3-methylpentanoic acid | Others | Not Available |
|  | 9.11 | 179.1 | 178.0506 | C_9_H_9_NO_3_ | [M-H]^-^ | 4-Acetamidobenzoic acid | [Benzene and substituted derivatives](http://classyfire.wishartlab.com/tax_nodes/C0002279) | Not Available |
|  | 11.69 | 120.06 | 119.0490 | C_8_H_8_O | [M-H]^-^ | Phenylacetaldehyde | Benzene and substituted derivatives | 122-78-1 |
|  | 16.13 | 352.2 | 353.2327 | C_20_H_34_O_6_ | [M+H]^+^ | Thromboxane B2 | [Fatty Acyls](http://classyfire.wishartlab.com/tax_nodes/C0003909) | 54397-85-2 |
|  | 11.58 | 205.1 | 206.0819 | C_11_H_11_NO_3_ | [M+H]^+^ | Indole-3-lactic acid | Indoles and derivatives | [1821-52-9](https://commonchemistry.cas.org/detail?cas_rn=1821-52-9) |
|  | 1.20 | 169.1 | 170.0928 | C_7_H_11_N_3_O_2_ | [M+H]^+^ | 1-Methylhistidine | Amino acids and their derivatives | 332-80-9 |
|  | 16.79 | 284.1 | 285.0765 | C_16_H_12_O_5_ | [M+H]^+^ | Wogonin | Flavonoids | 6665-74-3 |
|  | 16.14 | 316.2 | 317.2117 | C_20_H_30_O_4_ | [M+H]^+^ | 13,14-dihydro-15-keto Prostaglandin A2 | Fatty Acyls | 74872-89-2 |
|  | 13.33 | 236.07 | 237.0753 | C_12_H_12_O_5_ | [M+H]^+^ | 6,7,8-trimethoxy-2H-chromen-2-one | Heterocyclic Compounds | 6035-49-0 |
|  | 22.94 | 255.26 | 256.2635 | C_16_H_33_NO | [M+H]^+^ | N,N-Diethyldodecanamide | Amides | 3352-87-2 |
|  | 14.32 | 202.1 | 201.1132 | C_10_H_18_O_4_ | [M-H]^-^ | 3-tert-Butyladipic acid | Fatty Acyls | 10347-88-3 |
|  | 3.39 | 122.0 | 123.0448 | C_7_H_6_O_2_ | [M+H]^+^ | 4-Hydroxybenzaldehyde | Phenolic acids | 71-30-7 |
|  | 7.47 | 114.0 | 132.0813 | C_9_H_9_N | [M+NH_4_]^+^ | 6-Methylindole | Indoles and derivatives | 3420-02-8 |
|  | 16.39 | 316.2 | 317.2119 | C_20_H_30_O_4_ | [M+H]^+^ | Prostaglandin J2 | Fatty Acyls | 60203-57-8 |
|  | 7.02 | 194.1 | 217.1055 | C_11_H_12_N_4_O | [M+Na]^+^ | 6-(4-methoxyphenyl)pyrimidine-2,4-diamine | Pyrimidines | Not Available |
|  | 16.12 | 334.2 | 335.2221 | C_20_H_32_O_5_ | [M+H]^+^ | 15-keto Prostaglandin F2a | [Fatty Acyls](http://classyfire.wishartlab.com/tax_nodes/C0003909) | 35850-13-6 |
|  | 8.02 | 157.1 | 156.0660 | C_7_H_11_NO_3_ | [M-H]^-^ | 3-Methylcrotonylglycine | [Carboxylic acids and derivatives](http://classyfire.wishartlab.com/tax_nodes/C0000265) | 33008-07-0 |
|  | 12.39 | 192.0 | 193.0504 | C_10_H_8_O_4_ | [M+H]^+^ | Scopoletin | [Coumarins and derivatives](http://classyfire.wishartlab.com/tax_nodes/C0000145) | 92-61-5 |
|  | 15.87 | 278.1 | 279.0942 | C_18_H_15_OP | [M+H]^+^ | (11E,15Z)-9,10,13-trihydroxyoctadeca-11,15-dienoic acid | Fatty Acyls | 791-28-6 |
|  | 19.34 | 278.2 | 279.2323 | C_18_H_30_O_2_ | [M+H]^+^ | α-Eleostearic acid | [Fatty Acyls](http://classyfire.wishartlab.com/tax_nodes/C0003909) | Not Available |
|  | 12.651 | 286.05 | 285.04065 | C_15_ H_10_ O_6_ | [M-H]^-^ | Luteolin | [Flavonoids](http://classyfire.wishartlab.com/tax_nodes/C0000334) | 491-70-3 |
|  | 17.10 | 148.1 | 149.0964 | C_10_H_12_O | [M+H]^+^ | Cuminaldehyde | [Prenol lipids](http://classyfire.wishartlab.com/tax_nodes/C0000259) | 122-03-2 |
|  | 1.61 | 309.1 | 308.0996 | C_11_H_19_NO_9_ | [M-H]^-^ | N-Acetylneuraminic acid | [Organooxygen compounds](http://classyfire.wishartlab.com/tax_nodes/C0000323) | 19342-33-7 |
|  | 3.39 | 164.0 | 182.0819 | C_9_H_10_O_4_ | [M+NH_4_]^+^ | 3-(3,4-dihydroxyphenyl)propanoic acid | [Phenylpropanoic acids](http://classyfire.wishartlab.com/tax_nodes/C0002551) | 23028-17-3 |
|  | 16.97 | 194.13 | 195.1380 | C_12_H_18_O_2_ | [M+H]^+^ | Sedanolide | Benzofurans | 6415-59-4 |
|  | 1.53 | 142.1 | 143.1184 | C_7_H_14_N_2_O | [M+H]^+^ | Nitrosoheptamethyleneimine |  |  |
|  | 12.20 | 205.1 | 204.0667 | C_11_H_11_NO_3_ | [M-H]^-^ | Cinnamoylglycine | [Carboxylic acids and derivatives](http://classyfire.wishartlab.com/tax_nodes/C0000265) | 16534-24-0 |
|  | 1.64 | 244.1 | 243.0626 | C_9_H_12_N_2_O_6_ | [M-H]^-^ | Pseudouridine | [Nucleoside and nucleotide analogues](http://classyfire.wishartlab.com/tax_nodes/C0003737) | 1445-07-4 |
|  | 15.699 | 166.06 | 167.0703 | C_9_ H_10_ O_3_ | [M+H]^+^ | Apocynin | Phenolic compound | 498-02-2 |
|  | 1.51 | 243.1 | 266.0755 | C_9_H_13_N_3_O_5_ | [M+Na]^+^ | Cytidine | Nucleotides and their derivatives | 65-46-3 |
|  | 13.37 | 129.1 | 130.0658 | C_9_H_7_N | [M+H]^+^ | Isoquinoline | alkaloids | 119-65-3 |
|  | 14.42 | 262.1 | 261.0752 | C_13_H_14_N_2_O_2_S | [M-H]^-^ | ethyl2-[(3-methylbenzo[b]thiophen-2-yl)methylidene]hydrazine-1-carboxylate | Lignans and Coumarins | 1957-11-4 |
|  | 9.10 | 328.12 | 327.1089 | C_15_H_20_O_8_ | [M-H]^-^ | 4-Acetyl-3-hydroxy-5-methylphenyl β-D-glucopyranoside | Others | Not Available |

**Table S3. The targets of YWT**

| BRS3 | ITGAV | ITGB1 | ITGB5 | ITGB6 | JAK1 | JAK2 | JAK3 | FTO | GRIK1 |
| --- | --- | --- | --- | --- | --- | --- | --- | --- | --- |
| KDM3A | STS | THRA | ABAT | ALB | ALDH5A1 | CACNA1B | ADA | ADK | ADORA2B |
| ADORA3 | CCNA1 | CCNA2 | CDK2 | DAO | EPHX2 | GAPDH | GBA | GRK1 | HRAS |
| HSPA8 | IGFBP3 | MMP1 | MMP7 | MMP8 | OGA | PNP | PTPN2 | SLC28A3 | SLC5A1 |
| SLC5A2 | SLC5A4 | SRD5A1 | TDP1 | TYMP | GABRR1 | SLC6A11 | SLC6A13 | CCR8 | CYP2A6 |
| KIF11 | ROCK1 | ALDH2 | F10 | IL2 | NMUR2 | NQO2 | PDE5A | RPS6KA3 | SLC29A1 |
| TNF | ABAT | ACE | ALDH1A1 | ALPL | COMT | CSNK1A1 | CSNK1D | EP300 | ERN1 |
| HMGCR | MB | PLEC | CCKAR | KEAP1 | TRPA1 | ADRA1B | CASR | CCR3 | CHRNA2 |
| CHRNB4 | CXCR3 | DRD5 | EBP | GRIN2B | HTR5A | KCNN4 | MRGPRX1 | OPRK1 | OPRL1 |
| SLC18A3 | CYP2C19 | CYP2C9 | CYP3A4 | ERBB2 | HCAR2 | MAPK1 | SLC6A2 | SLC5A7 | APEX1 |
| BCHE | CALM1 | CBR1 | CYP1A1 | CYP1A2 | INSR | KIT | LCK | MAPT | MYLK |
| NAE1 | NTRK2 | OPRD1 | PIK3CG | PLA2G4A | SIGMAR1 | SLC22A12 | ST6GAL1 | TOP2A | PYGB |
| PYGM | ADAM17 | ADRB3 | ALDH3A1 | CCNE1 | CCNE2 | CLK3 | CYP2D6 | DRD1 | DYRK1B |
| FKBP1A | FLT1 | FNTA | GABRA1 | GALR3 | KCNA5 | MAPK10 | MAPK11 | MAPK8 | MAPK9 |
| MEN1 | PGGT1B | PPP1CA | RGS4 | RGS8 | RPS6KA2 | S1PR3 | TNFRSF1A | TRPC3 | TRPC6 |
| VCP | CRHR1 | DRD3 | DYRK1A | G6PC | GABRA1 | GABRA3 | KCNH2 | PDE2A | NOS1 |
| ADRB1 | ANPEP | EPHA1 | EPHA2 | EPHA3 | EPHA4 | EPHA5 | EPHA6 | EPHA7 | EPHA8 |
| EPHB1 | EPHB2 | EPHB3 | EPHB4 | EPHB6 | ERAP2 | HTR1B | HTR1D | HTR1E | HTR2A |
| HTR2C | HTR7 | IDO1 | MC1R | PIM2 | SLC15A1 | AMPD3 | CCND1 | CDK4 | CPA1 |
| F3 | FBP1 | FYN | KDM4C | NFE2L2 | RELA | SLC16A1 | TLR4 | TLR9 | TUBB1 |
| TUBB3 | FUT7 | LDHA | LDHB | SERPINE1 | SQLE | TPMT | ACLY | EGLN1 | ACACB |
| ASAH1 | HDAC2 | HDAC5 | HDAC8 | LAP3 | MTNR1A | MTNR1B | PDE3A | PDE3B | ROCK2 |
| TEK | ENPEP | GABBR2 | GFPT1 | GRIA1 | GRIA2 | GRIA4 | GRIK5 | HRH1 | HRH2 |
| HRH3 | HRH4 | KYNU | SLC6A1 | ACP1 | CD81 | CYP51A1 | DRD2 | GLUL | IL6 |
| MAPK3 | NR1H3 | PRKCH | PTGER1 | PTPN11 | PTPRF | RBP4 | TRPV1 | ABCB1 | ABCC1 |
| ABCG2 | ACHE | ADORA1 | ADORA2A | AHR | AKR1A1 | AKR1B1 | AKR1B10 | AKR1C1 | AKR1C2 |
| AKR1C3 | AKR1C4 | AKT1 | ALK | ALOX12 | ALOX15 | ALOX5 | AMY1A | APP | AR |
| ARG1 | AURKB | AVPR2 | AXL | BACE1 | CA1 | CA12 | CA13 | CA14 | CA2 |
| CA3 | CA4 | CA5A | CA6 | CA7 | CA9 | CAMK2B | CCNB1 | CCNB2 | CCNB3 |
| CD38 | CDK1 | CDK1 | CDK2 | CDK5 | CDK5R1 | CDK6 | CFTR | CSNK2A1 | CXCR1 |
| CYP19A1 | CYP1B1 | DAPK1 | DRD4 | EGFR | ESR1 | ESR2 | ESRRA | F2 | FLT3 |
| GLO1 | GPR35 | GRK6 | GSK3B | HSD17B1 | HSD17B2 | IGF1R | KDM4E | KDR | MAOA |
| MET | MMP12 | MMP13 | MMP2 | MMP3 | MMP9 | MPO | NEK2 | NEK6 | NOX4 |
| NUAK1 | PARP1 | PFKFB3 | PIK3R1 | PIM1 | PKN1 | PLA2G1B | PLG | PLK1 | PTGS2 |
| PTK2 | PTPRS | PYGL | SRC | SYK | TERT | TNKS | TNKS2 | TOP1 | TTR |
| TYR | XDH | ADH4 | ADH7 | ADRA1A | CHRNA3 | CHRNA4 | CHRNB4 | DNM1 | DNPEP |
| EZH2 | F2RL3 | GRM1 | HDAC3 | HSD17B7 | HTR1A | KISS1R | NAAA | NCOR2 | NPY5R |
| PAOX | PLA2G2C | RNPEP | SLC6A15 | SLC6A7 | SMO | TACR1 | TACR2 | KDM4A | KDM4B |
| KDM5B | FFAR4 | HTR2B | NR0B2 | PTGER4 | PTPRC | RORA | RORB | SAE1 | UBA2 |
| ADH1A | ADH1B | ADH1C | CES1 | CHRM1 | CHRM2 | CHRM3 | CHRM4 | CHRM5 | CHRNA4 |
| CHRNA7 | CHRNB2 | MCL1 | ACE2 | ADAM12 | APLNR | BIRC2 | BMP1 | BRD4 | CAPN1 |
| CAPN1 | CAPN2 | CAPNS1 | CASP1 | CASP3 | CASP6 | CASP7 | CASP8 | CASP9 | CCR1 |
| CELA1 | CNR2 | CPB1 | CPB2 | CTRB1 | CTRC | CTSD | CXCR2 | DPP4 | DPP7 |
| DPP8 | DPP9 | ECE1 | EDNRB | FUCA1 | HCRTR1 | HCRTR2 | HDAC1 | HDAC4 | HDAC6 |
| ITGA2 | ITGA2B | ITGAV | ITGB1 | ITGB3 | KDM6B | KLK3 | LGMN | LPAR2 | NFKBIA |
| NTSR1 | PLAU | PPIA | PREP | PSMB1 | PSMB2 | PSMB5 | PTGDR | PTGER3 | SIRT1 |
| SIRT2 | SIRT3 | TACR3 | TBXA2R | TGM1 | TGM2 | TPSAB1 | XIAP | ADRA2A | ADRA2B |
| ADRA2C | ATP12A | BCL2L1 | CCR5 | CDC25A | CDC25B | CDC25C | CES2 | CREBBP | CTSB |
| CTSC | CTSF | CTSH | CTSK | CTSL | CTSS | CTSV | CYP11B1 | CYP11B2 | CYP17A1 |
| DNMT3A | ELANE | EPHX1 | FNTA | FNTB | GABRA2 | GABRA5 | GABRB2 | GABRB3 | GABRG2 |
| GABRG2 | GPR55 | GRM5 | HSD11B1 | HSD17B3 | HTR6 | IARS | ICAM1 | IKBKB | IL1B |
| KCNK2 | MAOB | MAPK14 | MGLL | MIF | NOS2 | P2RX7 | PABPC1 | PCSK7 | PDCD4 |
| PDE10A | PDE4D | PDE7A | PGR | PIK3CA | PIK3CB | PIK3CD | PLA2G2A | POLA1 | POLB |
| PPARA | PPARD | PRKCA | PRKCD | PRKCE | PRKDC | PRSS1 | PTGS1 | PTPN1 | RAPGEF4 |
| RPS6KA5 | SELE | SHBG | SLC6A3 | SLC6A4 | SRD5A2 | STAT3 | TAS2R31 | TBXAS1 | THRB |
| TTL | VCAM1 | XPO1 | CACNA2D1 | CDC45 | CPA3 | FABP2 | FDFT1 | FDPS | FOLH1 |
| G6PD | GABBR1 | GPBAR1 | GSTK1 | HAO1 | HSD11B2 | KDM2A | KDM5C | KMO | LTA4H |
| NPC1L1 | NR1H4 | PHF8 | PTGER2 | PTGFR | REN | SERPINA6 | SLC22A6 | UGT2B7 | CA5B |
| NEU4 | PDE1B | PDE9A | PTPN22 | SLC37A4 | DDO | HSP90AA1 | HSP90B1 | MPG | ODC1 |
| OPRM1 | ABHD6 | AGTR1 | ALOX5AP | APH1A | APH1B | BCL2 | CCKBR | CMA1 | CNR1 |
| CPT1A | CPT1B | CTSA | CTSG | CYP26A1 | CYP26B1 | DAGLA | DAGLB | EDNRA | ENPP2 |
| FAAH | FABP1 | FABP3 | FABP4 | FABP5 | FFAR1 | GCG | GLRA1 | GRM2 | HNF4A |
| ICAM1 | ITGAL | ITGAL | ITGB2 | LTB4R | MDM2 | MME | NCSTN | NR1H2 | NR3C1 |
| NR3C2 | OXER1 | PDE4A | PDE4B | PDE4C | PLA2G10 | PPARG | PRKAA2 | PRKAB1 | PRKAG1 |
| PSEN1 | PSEN1 | PSEN2 | PSENEN | PTGDR2 | PTGES | PTGES2 | PTGIR | PTPN6 | RARA |
| RARB | RARG | RORC | RXRA | RXRB | RXRG | SCD | TP53 | TRPM8 | TSPO |
| VDR | SQSTM1 | MAP1LC3A |  |  |  |  |  |  |  |

**Table S4. The targets of cisplatin-induced AKI**

| TP53 | MET | ACE | IL6 | TNF | PTEN | NPM1 | PAX2 | MTOR | KIT |
| --- | --- | --- | --- | --- | --- | --- | --- | --- | --- |
| MIR21 | CRP | IFNG | PIK3CA | TERT | STAT3 | ALB | CDKN2A | AGT | MEG3 |
| TGFB1 | TUG1 | EGF | JAK2 | KRAS | KMT2A | RET | BAX | LCN2 | SETD2 |
| IL1B | ATM | PTPN11 | MIR126 | EPO | MIR155 | H19 | NFE2L2 | BDNF-AS | IL2 |
| VEGFA | CASP3 | HMOX1 | MPO | MIR125A | CCL2 | B2M | TLR4 | HOTAIR | TLR3 |
| F2 | MIR221 | MIRLET7C | GAS5 | ICAM1 | ABCB1 | MYC | BCR | BCL2 | MMP9 |
| SOD2-OT1 | CST3 | XIST | HIF1A | EGFR | MAPK1 | CERNA3 | ABL1 | TRAF3 | CCND1 |
| MIR142 | NLRP3 | MIR320A | AKT1 | EDN1 | MIR23A | NOS2 | LINC01672 | MIR335 | MIR146A |
| CTNNB1 | AQP2 | CBFB | UCA1 | SOD1 | IL18 | SERPINE1 | C3 | PGR-AS1 | CCAT1 |
| MTHFR | KCNQ1OT1 | TET2 | SETBP1 | CDKN2B-AS1 | MIR222 | BAP1 | NF1 | FANCD2 | LINC02605 |
| FAS | XDH | SRSF2 | PTGS2 | IFNA1 | F3 | NEAT1 | MB | MIR199B | OGG1 |
| MUC1 | IL1RN | CAT | NOTCH1 | NAGLU | HGF | IGF1 | ERBB2 | FBXW7 | MIR204 |
| MIR29A | HPRT1 | PARP1 | CDKN1A | CFTR | SLC2A1 | MIR34C | MIR27B | MIR494 | MIRLET7E |
| LEP | TMX2-CTNND1 | SPP1 | SLC12A1 | BDNF | MDM2 | IL3 | CYCS | RRM2B | HMGB1 |
| TF | MAPK8 | GSTM1 | FASLG | NFKB1 | LRP2 | CXCR4 | PDGFRB | CASP8 | PPARG |
| CASP9 | ABCG2 | GSTP1 | EPOR | CDH1 | FGF2 | PIK3CG | NBAS | LPP | CHEK2 |
| MIR34A | JUN | BCL2L1 | TNFRSF1A | TLR2 | MMP2 | CD274 | CDKN1B | CCL5 | HFE |
| EZH2 | SLC17A5 | GFRA1 | LDHA | MIR145 | MIF | AMBP | STAT1 | MIR141 | LINC-ROR |
| AQP1 | MAPK3 | BIRC5 | MIR203A | FOS | MYB | MIR199A1 | WNT4 | TP73 | MAPK14 |
| LGALS3 | COMT | ABCC1 | ESR1 | IL7 | VDR | SOD2 | FABP1 | CD40 | MIR16-1 |
| MIR146B | CXCL12 | HADHA | CAV1 | PLG | ANXA5 | SRC | XIAP | CCN2 | HNF4A |
| TNFSF10 | BRCA1 | MAP2K1 | MT-TL1 | IGF2 | GGT1 | ALDH2 | MSH2 | PCNA | CDK4 |
| CASC2 | CD44 | IFNA2 | GHRL | GSTT1 | SIRT1 | NTRK1 | PTGS1 | GAPDH | MIR134 |
| MIR106B | CLU | MIR133B | DNMT1 | PRL | MIR372 | MIR7-3HG | ERCC4 | ITGB3 | RELA |
| MIR214 | MCL1 | ABCC2 | GSK3B | MIR144 | STAT5A | LMNA | PIK3CB | TUSC7 | JAG1 |
| MMP3 | SLC22A2 | CDK2 | TBL1XR1 | RAF1 | TRA-TGC7-1 | GSN | MIR708 | PWAR1 | HSP90AA1 |
| FGFR2 | MAPK10 | TPMT | CASP1 | CA9 | MIR93 | ALOX5 | RAD51 | HSPB1 | NQO1 |
| RUNX2 | SP1 | XRCC1 | PPARA | NOS1 | DANCR | MIR216A | EGR1 | RARB | ITGB1 |
| CDKN3 | HSPA5 | NR3C1 | MYCN | GSR | F10 | TRAP1 | GJA1 | LTF | FOXO3 |
| KRT18 | SET | MIR10B | TNFRSF1B | CDK1 | TKT | S100A9 | DMD | MIR125B1 | SFPQ |
| IL11 | SOX9 | NOX4 | IGFBP2 | NFKBIA | TAC1 | NONO | S100A8 | RB1 | TXN |
| DPEP1 | EMSLR | YAP1 | SNHG7 | MVP | ITGAL | TERC | AURKA | ACHE | SQSTM1 |
| KRT8 | DRD2 | BCL2L11 | MIR98 | CEBPB | HSPA1A | BECN1 | DNTT | PRKCD | GJB1 |
| DIS3L2 | WFS1 | APC | MIR423 | IRF1 | EIF4EBP1 | GLI1 | FANCL | DNAH8 | TOP2A |
| TNFRSF10B | LUCAT1 | TYMS | MIR181A1 | MIR20A | LIF | AR | PRKCA | MIR191 | ATP7B |
| PPARGC1A | CHUK | MIR29B1 | PLA2G6 | LYN | HSPD1 | DICER1 | RPS27A | HTR2A | FLNA |
| BSG | CASP2 | E2F1 | PROM1 | SMPD1 | HDAC1 | ERBB3 | MLH1 | PRKACA | RPS6KB1 |
| AKT3 | CDKN1C | PRSS1 | SNORD15A | IL18R1 | CCNA2 | MIR127 | HSPA8 | MIR139 | NTRK2 |
| DHFR | PKM | TRPV1 | CDK6 | ERCC2 | PIK3R1 | CASP7 | HDAC6 | AKR1B1 | PMS2 |
| NR1I2 | NR4A1 | RMRP | CFLAR | ASS1 | LINC00473 | ALDOB | HTR3A | APAF1 | FOXM1 |
| NGFR | BAK1 | PODXL | MSH6 | TGFBR1 | BMI1 | ADAM17 | VEGFC | HBEGF | PTK2B |
| TIMP3 | ATRIP | ALDOA | RAC1 | SMAD2 | CHEK1 | MIR211 | TH | KRT5 | MIR31 |
| RNY3 | UGT1A1 | ATF3 | PRPS1 | IFI27 | H2AX | PRKDC | ZEB1 | CTNNA1 | ENO1 |
| DIABLO | VTN | TACR1 | SCARNA5 | SGPL1 | ADD1 | PTK2 | MIR26A1 | AFDN | IKBKB |
| CCNB1 | POU5F1 | ANXA2 | BAD | SLC5A1 | LBR | RAB4B-EGLN2 | ERCC1 | EIF4E | RPS19 |
| APEX1 | ABCB11 | SNAI1 | MIR212 | PRKN | COX5A | PARK7 | CTSL | ZEB2 | GLUD1 |
| SOX2 | CCND2 | AIFM1 | HDAC8 | MIR485 | TP63 | HTR1A | PLK1 | RARS1 | MEG8 |
| PRKCB | SMAD7 | TOP1 | PEX1 | NR1H4 | MMP7 | CNR1 | CCN1 | KLF4 | TRC-GCA24-1 |
| ACP1 | CCND3 | DBH | RASA1 | ITGA6 | DDIT3 | ATF2 | NES | NME1 | MIR101-1 |
| MT-RNR1 | DNM1L | VDAC1 | RNLS | ABCA1 | FADD | FASN | MIR425 | STK11 | STING1 |
| STUB1 | SLC5A6 | HAX1 | CUL3 | NTN1 | EIF2AK2 | FANCF | MIR215 | DUSP1 | MIR362 |
| WRN | EZR | TYMP | TRE-TTC3-1 | HK2 | UCP2 | MIR183 | TGM2 | RPL11 | MST1R |
| TNFRSF10A | ADCYAP1 | SNHG1 | BIRC2 | SHC1 | ATP7A | PWAR4 | AKT2 | XRCC3 | BLZF1 |
| SOS1 | IRS1 | GPX1 | CCR7 | HES1 | ADAM10 | NTRK3 | VTRNA1-1 | MT-TS1 | KCNN4 |
| TET1 | SLC22A1 | ARNT | DUSP6 | TRAF2 | RNU6-1 | PCAT1 | SLC9A1 | GPI | HNMT |
| CYLD | MDM4 | EGLN1 | MIR885 | BNIP3 | XBP1 | MIR193B | LAMP1 | HTRA1 | KEAP1 |
| XRCC2 | PDCD1LG2 | LINC01554 | RPS6 | NANOG | ABCC5 | CHKB-CPT1B | ABCC3 | PLA2G4A | MIR148B |
| ESR2 | MIR125B2 | PRDX6 | CASP10 | FOXP2 | CAMK2G | SNHG16 | BIRC3 | GADD45A | C1QBP |
| CYP1B1 | HULC | MAD2L2 | SLC15A2 | SNORD118 | ATF4 | CASP6 | ATP5F1A | MIR137 | JUP |
| BBC3 | EPHX1 | EIF2S1 | MCAM | MIR625 | AURKB | MIR381 | BID | RIPK3 | TBXA2R |
| MAP3K5 | PRDX5 | PTPA | FABP4 | DPYD | MIR197 | BCL2L2 | CLCN3 | NRG1 | HSP90AB1 |
| HOXA13 | PLCG1 | ATR | SLC6A6 | MIR363 | CNOT3 | LAMB3 | PGR | NDUFS3 | RPA1 |
| NCL | MIP | SIRPA | VNN1 | PRDX1 | PRKAA1 | LDHB | CRYAB | SKP2 | MST1 |
| KRT10 | CNR2 | MIR32 | PRMT5 | RPL15 | BMAL1 | MDH2 | MRE11 | MMP13 | SPHK1 |
| MIR500A | KAT5 | MAPK9 | HNRNPA2B1 | RIPK1 | ADD3 | TPI1 | MIR373 | MIR138-1 | SIRT3 |
| DYRK1A | MAP2K3 | PPIA | SIRT2 | XPA | SLC31A1 | STIM1 | VEGFD | ERCC6 | MIR24-2 |
| XRCC5 | PTPN3 | RPS7 | PMAIP1 | MGMT | MIR503 | GLO1 | PEBP1 | ASNS | CCNC |
| AREG | TNFSF13 | YWHAE | PLEK | MAP3K1 | UGT1A9 | H2BC21 | MIR379 | TRIM21 | C1R |
| JUND | SNAI2 | WEE1 | UGT2B7 | USP9X | GSDMD | MIR205 | OIP5-AS1 | PRKG1 | S100A4 |
| MIR302A | SPTBN1 | ASAH1 | KIF4A | PPM1D | HNRNPK | TMPO | TRPA1 | TWIST1 | NSUN2 |
| XPC | RPL35 | PSMC4 | XRCC6 | MSH3 | HERC2 | CSNK2A1 | MIR509-1 | FAS-AS1 | KLF5 |
| TUBB | ADH1B | ATG5 | SNAP29 | BAG1 | HOXA11-AS | ITCH | CFL1 | DLEU1 | TFAM |
| NOX1 | MIR501 | CLDN1 | CAPN1 | HSPB2 | RECK | CGAS | KDM4C | EPHA2 | ZFP36 |
| HSPA9 | CDH17 | DLEU2 | TP53BP2 | MMP10 | CGB5 | PSTPIP2 | NEDD9 | DUSP19 | EIF4G2 |
| SOX4 | YBX1 | UGT1A6 | MUC3A | DLD | HNRNPU | CDC25A | C1QTNF3 | RBM5 | PIDD1 |
| PRDX3 | DDB1 | MALT1 | PTBP1 | GAS1 | ID3 | PRKCH | POLR1C | CCNG1 | UBE2L3 |
| MT3 | ULK1 | BST2 | GSTM3 | RPLP2 | MT1E | KHDRBS1 | ADH1C | ENDOG | PAK1 |
| TPH1 | DHX9 | DLST | AOX1 | UFL1 | ID1 | PGD | FBP1 | NFAT5 | SSRP1 |
| ST3GAL3 | HTRA2 | SRPK1 | CXCL17 | RCAN1 | SPHK2 | MLKL | RPS6KA1 | LINC00472 | LINC01191 |
| PRKCQ | YWHAQ | NCF2 | KPNA2 | SYVN1 | PDCD4 | MIR375 | NFYA | HMGA1 | MAP3K8 |
| MIR136 | RRAS2 | UGT1A7 | FGF19 | SIRT5 | TIGAR | SLC16A1 | RPL22 | PRKAB1 | CCN3 |
| LIG1 | ARSK | GABBR2 | GCLC | CARD8 | S100A6 | PLS3 | ATL1 | PFN1 | SLC47A1 |
| PABPC1 | PTAFR | POLR2L | ITPR1 | NEDD4 | DELEC1 | YWHAB | REL | BRAP | ADAM9 |
| CEBPD | PPT1 | RAD50 | IGHG1 | UBC | STIP1 | LILRB4 | S1PR2 | PURA | FOSL1 |
| SLC11A2 | TXNRD1 | STK4 | MIR135B | MSX1 | IVNS1ABP | TUBA1B | TRPM2 | TAB2 | HELLS |
| PLSCR1 | HM13 | REV3L | CCT6A | CYB561D2 | LRIG1 | CDC20 | UBE4B | UGT1A4 | RNY5 |
| SCD | DSPP | PLAA | RIPK4 | MIR302C | NDRG2 | SSB | KCNH1 | CD5L | MAD2L1 |
| HNRNPL | OGDH | RAD9A | BAG3 | PPP1R15A | UACA | PRMT1 | PRPS2 | MIR33A | CYP4A11 |
| MGA | MAP2K6 | POLB | TLN1 | MIR449A | TRIB1 | FLOT1 | PAWR | GSK3A | YWHAZ |
| ERCC3 | HUS1 | RORC | CARS2 | CAPRIN1 | RPS16 | SIVA1 | PDK1 | FERMT3 | PDE3A |
| TLK1 | TNFAIP2 | FFAR1 | CYP20A1 | SATB1 | SLC4A7 | MIR874 | BCYRN1 | GSDME | HTRA3 |
| BCL2L12 | ATG14 | CBX3 | ADH7 | ISL1 | BANF1 | ICAM2 | PIWIL4 | BIK | ALDH4A1 |
| EEF2 | BHLHE40 | CARD10 | ATP5F1B | COP1 | LINC01234 | FBLN7 | PDCD5 | MIR33B | TMEM33 |
| PPP1R13L | CUL1 | YWHAG | CHM | PRDX4 | ARL6IP1 | TXN2 | PPP2R2A | GSTO1 | CSNK1A1 |
| DYNLL1 | CDH3 | ING1 | IL17B | CDC25C | OGT | COL11A1 | MAPK11 | MIR1184-1 | TRIM25 |
| ANXA3 | DDX5 | MIR16-2 | UTRN | MMP19 | CLCN6 | CSN2 | PDIA6 | ATP2C1 | G3BP1 |
| MIR519D | PPP4C | RBM4 | MIR3168 | ST20 | RIF1 | VTRNA1-2 | REV1 | DCD | HSPE1 |
| COPS5 | RPS15 | AKAP13 | ZBTB7A | DGCR2 | NARS1 | PGRMC1 | TRP-AGG2-5 | POLI | UGT8 |
| MT-RNR2 | TAGLN2 | RPS3A | SPAAR | MAB21L1 | FZD7 | PDE1A | GAN | DEFB103B | UGT1A3 |
| HEXD | PTPRS | CAMK2A | ACVR1B | STOML2 | SND1 | PRPSAP1 | PPIB | SULF1 | RPS4X |
| DDB2 | PDIA4 | ANKRD1 | SPTBN5 | MIR505 | POLH | LINCMD1 | UBE2C | BASP1 | RPA2 |
| S100A16 | TRIM31 | PMPCB | FMO2 | PSME3 | AMBRA1 | PRPSAP2 | TRIM37 | MIR1294 | FMO3 |
| MT4 | GOSR1 | ARSH | LRRC8A | MAP2K7 | DCAF1 | TP53TG1 | VTRNA2-1 | RAD1 | HNRNPM |
| SCRIB | DTNB | ABCG1 | BPTF | SULF2 | RPS11 | TRIM44 | PNPLA8 | FMO5 | RAB1A |
| ATP6V1C1 | DDX17 | RPS3 | HNRNPDL | ASCL1 | MYL6 | GPR37 | TANK | PARP4 | TXNL1 |
| COIL | CYP4B1 | MIR655 | PRNCR1 | MYDGF | TMED10 | PRLR | CERS6 | HNRNPAB | USP4 |
| SPA17 | RAD23A | E2F3 | SMG1 | MYBBP1A | LINC01614 | JCHAIN | DYRK1B | CDC34 | CNTN1 |
| PGRMC2 | OTUB1 | PRSS3 | PSMD10 | SLC39A4 | RNF114 | OGFR | EIF3G | TRIM47 | GSTM4 |
| AKR1C1 | ATG4B | MIR3182 | TMED1 | SYNCRIP | SIX3-AS1 | SEL1L | AKR1C2 | MZF1 | FOXC2-AS1 |
| ARHGDIB | POLR1G | MAP1LC3A |  |  |  |  |  |  |  |

**Table S5. The key targets of YWT in cisplatin-induced AKI**

| ITGB1 | JAK2 | ALB | CCNA2 | CDK2 | GAPDH | HSPA8 | MMP7 | SLC5A1 | TYMP |
| --- | --- | --- | --- | --- | --- | --- | --- | --- | --- |
| ALDH2 | F10 | IL2 | TNF | ACE | COMT | CSNK1A1 | MB | KEAP1 | TRPA1 |
| KCNN4 | ERBB2 | MAPK1 | APEX1 | KIT | NTRK2 | PIK3CG | PLA2G4A | TOP2A | ADAM17 |
| DYRK1B | MAPK10 | MAPK11 | MAPK8 | MAPK9 | TNFRSF1A | DYRK1A | NOS1 | EPHA2 | HTR2A |
| CCND1 | CDK4 | F3 | FBP1 | KDM4C | NFE2L2 | RELA | SLC16A1 | TLR4 | LDHA |
| LDHB | SERPINE1 | TPMT | EGLN1 | ASAH1 | HDAC8 | PDE3A | GABBR2 | ACP1 | DRD2 |
| IL6 | MAPK3 | PRKCH | PTPN11 | TRPV1 | ABCB1 | ABCC1 | ABCG2 | ACHE | AKR1B1 |
| AKR1C1 | AKR1C2 | AKT1 | ALOX5 | AR | AURKB | CA9 | CCNB1 | CDK1 | CDK6 |
| CFTR | CSNK2A1 | CYP1B1 | EGFR | ESR1 | ESR2 | F2 | GLO1 | GSK3B | MET |
| MMP13 | MMP2 | MMP3 | MMP9 | MPO | NOX4 | PARP1 | PIK3R1 | PLG | PLK1 |
| PTGS2 | PTK2 | PTPRS | SRC | TERT | TOP1 | XDH | ADH7 | EZH2 | HTR1A |
| TACR1 | ADH1B | ADH1C | MCL1 | BIRC2 | CAPN1 | CASP1 | CASP3 | CASP6 | CASP7 |
| CASP8 | CASP9 | CNR2 | HDAC1 | HDAC6 | ITGB3 | NFKBIA | PPIA | SIRT1 | SIRT2 |
| SIRT3 | TBXA2R | TGM2 | XIAP | BCL2L1 | CDC25A | CDC25C | CTSL | EPHX1 | ICAM1 |
| IKBKB | IL1B | MAPK14 | MIF | NOS2 | PABPC1 | PDCD4 | PGR | PIK3CA | PIK3CB |
| POLB | PPARA | PRKCA | PRKCD | PRKDC | PRSS1 | PTGS1 | STAT3 | NR1H4 | UGT2B7 |
| HSP90AA1 | BCL2 | CNR1 | FABP1 | FABP4 | FFAR1 | HNF4A | ITGAL | MDM2 | NR3C1 |
| PPARG | PRKAB1 | RARB | RORC | SCD | TP53 | VDR | SQSTM1 | MAP1LC3 |  |
